# Identification of candidate lncRNAs and circRNAs regulating WNT3/β-catenin signaling in essential hypertension

**DOI:** 10.18632/aging.103137

**Published:** 2020-05-11

**Authors:** Liang Yin, Jie Yao, Guangxue Deng, Xuemei Wang, Weijuan Cai, Jie Shen

**Affiliations:** 1 Department of Endocrinology, Shunde Hospital of Southern Medical University, Shunde 528300, China; 2 Shunde Hospital of Southern Medical University, Shunde 528300, China; 3 State Key Laboratory of Bioelectronics, Chien-Shiung Wu Lab, School of Biological Science and Medical Engineering, Southeast University, Nanjing 210096, China

**Keywords:** long noncoding RNA, circular RNA, microRNA, epigenetics, essential hypertension

## Abstract

Mounting evidence suggests that noncoding RNAs (ncRNAs) contribute to the pathogenesis of cardiovascular diseases. However, their role in essential hypertension (EH) is still unclear. We therefore identified differentially expressed long noncoding RNAs (lncRNAs) and circular RNAs (circRNAs) in EH patients from a high-risk population group and constructed a competing endogenous RNA regulatory network that predicts interactions of potential diagnostic and therapeutic relevance between specific lncRNA/circRNA-microRNA-mRNA triplets. Our analysis identified two lncRNAs, transmembrane protein 183A pseudogene (LOC646616) and leucine aminopeptidase 3 pseudogene 2 (LAP3P2), and two circRNAs, hsa_circ_0039388 and hsa_circ_0038648, that are highly co-expressed with both wingless-type MMTV integration site family member 3 (WNT3) and calcium/calmodulin-dependent protein kinase II inhibitor 2 (CAMK2N2) mRNAs and also share common microRNA binding sites with these two transcripts. We also confirmed that a mutually regulated network composed of LOC646616/microRNA-637/WNT3 controls WNT3 expression and influences viability and invasive properties in human arterial smooth muscle cells in vitro. These findings highlight a novel ncRNA-based regulatory mechanism potentially driving WNT/β-catenin activation in EH, and suggest that the identified ncRNAs may represent useful biomarkers and therapeutic targets for this condition.

## INTRODUCTION

Hypertension affects ~26% of adults worldwide and its prevalence is expected to increase to 29%, encompassing 1.56 billion people, by 2025 [1]. Essential hypertension (EH) is defined as a chronic elevation in blood pressure with no discernible cause. EH accounts for about 95% of all hypertension cases and is a major risk factor for coronary heart disease, stroke, and kidney failure. Despite being a major public health problem, the etiology and pathogenesis of EH remain incompletely understood.

Xinjiang is a multi-ethnic region with diverse living habits and distinct prevalences and characteristics defining various chronic diseases such as EH, type 2 diabetes, and esophageal and cervical cancers. An epidemiological investigation has shown that Xinjiang’s Kazakhs constitute a high-risk population for EH, as the prevalence of EH in Kazakh residents >45 year-old can be as high as 53.6%, which is significantly higher than that reported for the Uygur and Han populations in the same area [2]. EH is a complex disease influenced by interactions between genetic and environmental factors. A stronger role for lifestyle/environmental factors seems apparent given that a single risk allele can only explain increases in systolic and diastolic blood pressure of 0.5-1.0 mmHg, while to date genome-wide association studies could overall attribute less than 2% of blood pressure changes to genetic variation [3].

Non-protein-coding RNAs (ncRNAs), including long noncoding RNAs (lncRNAs), circular RNAs (circRNAs) and microRNAs (miRNAs), have emerged as important regulators of gene transcription and protein translation that influence both normal physiology and major chronic diseases such as cancer, atherosclerosis, and type 2 diabetes [4–6]. The advent of high-throughput RNA sequencing (RNA-Seq) technology and bioinformatics tools has allowed the identification of thousands of ncRNAs expressed in various organisms. In parallel, studies addressing the expression of disease-specific ncRNAs highlighted their potential as novel diagnostic and prognostic biomarkers. However, the contribution of ncRNA species to the onset and development of hypertension is still incompletely characterized. Various studies have documented that lncRNAs, circRNAs, and miRNAs can be detected in bodily fluids such as serum, plasma, urine, saliva, and within exosomes [7, 8]. This property enhances the potential of ncRNAs to serve as non-invasive markers of disease. The recently advanced competing endogenous RNA (ceRNA) hypothesis proposes that various RNA species (including lncRNAs, circRNAs, pseudogenes, and mRNAs) sharing common miRNA response elements (MREs) regulate other RNA transcripts by competitive binding to the same miRNA [9]. To date, hundreds of putative regulatory ceRNA networks typically composed of lncRNA/circRNA-miRNA-mRNA triplets have been identified through bioinformatics tools and many have been validated by cellular assays [10, 11].

Wingless-type MMTV integration site (WNT) signaling plays an important role during cardiogenesis by directing mesoderm formation, cardiac progenitor cell specification and proliferation, and angiogenesis [12]. Whereas its activity subsides in the healthy adult cardiovascular system, canonical WNT signaling through β-catenin (i.e. WNT/β-catenin pathway) is reactivated in hypertension and contributes to various heart conditions including fibrosis, hypertrophy, arrhythmias, and infarction [13]. WNT family member 3 (WNT3) is a potent activator of the canonical WNT pathway [14]. A positive-feedback loop has been described by which β-catenin occupies the WNT3 promoter in a WNT3-dependent manner to activate WNT3 expression and sustain WNT3/β-catenin signaling [15]. Calcium/calmodulin-dependent protein kinase II (CAMKII) is also a key signaling molecule in cardiovascular disease, whose activity is endogenously inhibited by two recently identified proteins termed CAMK2N1 and CAMK2N2 [16]. Interestingly, activation of CAMKII through the noncanonical WNT/Ca^2+^ pathway was shown to antagonize canonical WNT/β-catenin signaling via activation of the TAK1-NLK MAPK pathway [17, 18]. Although numerous ncRNAs were shown to regulate WNT/β-catenin signaling in various cell types [19–21], and a few lncRNAs and miRNAs were recently linked to coronary heart disease and hypertension [22], the potential regulation of WNT3 and CAMK2N2 expression by ncRNAs in EH remains undefined.

Thus, our study aimed to comprehensively identify differentially expressed (DE) lncRNAs and circRNAs in Kazakh individuals with EH, and to unmask potential miRNA-WNT3/CAMK2N2-based ceRNA regulatory networks.

## RESULTS

### Identification of differentially expressed lncRNAs and circRNAs in hypertensive patients

**Figure 1:**
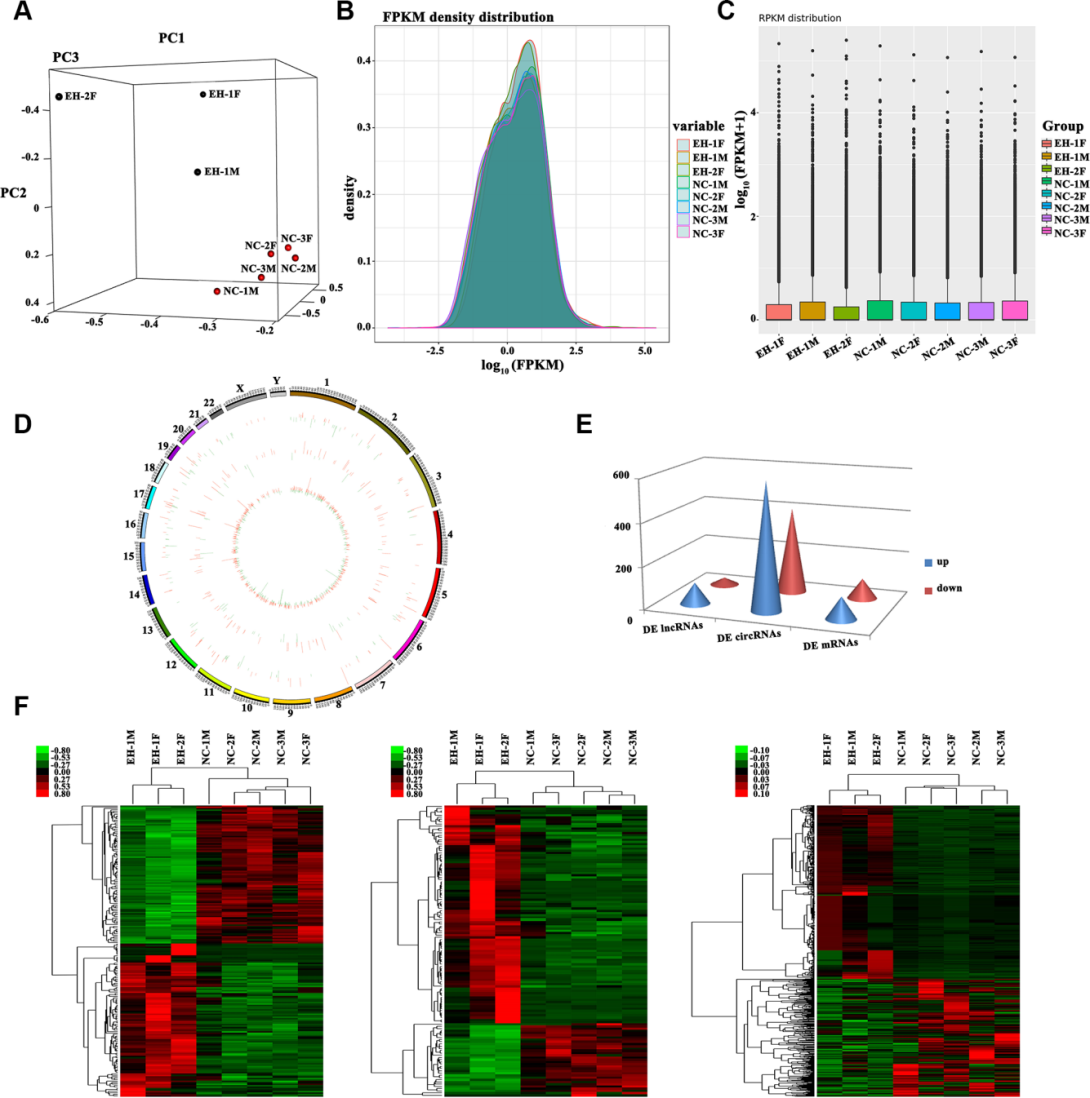
**Profiling of differentially expressed RNAs between hypertensive subjects and normotensive controls.** (**A**) 3D Principal Component Analysis (3D-PCA) showing the distribution of sequenced samples from hypertensive and normotensive subjects. (**B**) Boxplots of FPKM values (log_10_-scale) for different samples indicating the distribution of these values about the median. (**C**) FPKM density distribution for each sample. Different colors represent unique samples. (**D**) LncRNAs and circRNAs were broadly distributed across the 24 pairs of human chromosomes. Upregulation is indicated in red and downregulation is shown in green. Height indicates degree of difference in expression. (**E**) Quantification of DE lncRNAs, circRNAs, and mRNAs. (**F**) Heat maps based on the expression values of DE (log_2_ FC>1 or <-1, FDR<0.05) lncRNAs, circRNAs, and mRNAs. Expression values are represented by a color scale; red indicates high relative expression, and green indicates low relative expression. Each column represents one sample, and each row indicates a specific transcript.

An initial high-throughput RNA-seq analysis was performed at Novel Bioinformatics Ltd., Co., Shanghai for detection of DE lncRNAs and circRNAs in peripheral blood mononuclear cells (PBMNCs) from 3 hypertensive subjects and 5 normotensive individuals (Supplementary Table 1), all members of the Kazakh population of Xinjiang, northwest China. Principal component analysis (PCA) revealed that different expression profiles distinguished hypertensive from normotensive subjects (Figure 1A). For all genes, normalized fragments per kilobase per million reads (FPKM) densities were similar between hypertension and normotension samples (Figure 1B, 1C). The number and distribution of mRNAs, lncRNAs, and circRNAs are displayed in a volcano plot (Supplementary Figure 1A–1C). The distribution of lncRNAs and circRNAs on the 24 pairs of human chromosomes were presented on Circos plot (Figure 1D).

Screening for DE lncRNAs, circRNAs, and mRNAs was based on log_2_ fold change (FC)> 1 or <-1 and false discovery rate (FDR) <0.05 for the RNA-Seq data. Results yielded 131 DE lncRNAs (98 upregulated and 33 downregulated), 1,002 DE circRNAs (597 upregulated and 405 downregulated) and 200 DE mRNAs (105 upregulated and 95 downregulated) (Figure 1E). Hierarchically-clustered heat maps for the above DE RNAs are shown in Figure 1F.

### Gene ontology and KEGG pathway analyses

To predict the potential functional implication of the identified DE RNAs, we performed Gene Ontology (GO) and Kyoto Encyclopedia of Genes and Genomes (KEGG) functional enrichment analyses. GO analysis results for the top 15 DE mRNAs are shown in Supplementary Figure 2A. DE lncRNAs were mainly enriched in ‘phosphorylation’, ‘large ribosomal subunit’, and ‘transferase activity, transferring phosphorus-containing groups’ (Supplementary Figure 2B), while DE circRNAs were mainly enriched in ‘protein phosphorylation’, ‘nucleoplasm’, and ‘protein binding’ (Supplementary Figure 2C). KEGG pathway analysis showed that the DE mRNAs were significantly enriched in pathways related to non-alcoholic fatty liver disease (NAFLD), glycerophospholipid metabolism, TNF signaling, and WNT signaling (Supplementary Figure 2D, 2E), which are all related to inflammatory processes and dyslipidemia. Meanwhile, DE circRNAs were considerably enriched in pathways related to RNA degradation, WNT signaling, and MAPK signaling (Supplementary Figure 2F), all of which have been linked to hypertension. In contrast, we found no pathways in association with our DE lncRNA set in the KEGG database.

### Gene co-expression network

**Figure 2:**
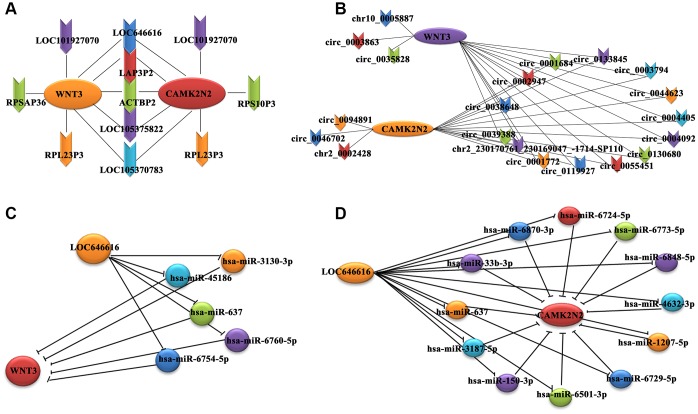
**Co-expression subnetwork of DE lncRNAs associated with WNT3 and CAMK2N2.** (**A**) Co-expression network analysis indicating positive correlation between 7 DE lncRNAs and both WNT3 and CAMK2N2. (**B**) A positive correlation with both WNT3 and CAMK2N2 was detected for 14 circRNAs. We used the circBase database for gene annotation; only one gene was not annotated in circBase. (**C**, **D**) A miRNA-based sponge regulatory subnetwork depicting LOC646616/miRNA/WNT3 and LOC646616/miRNA/CAMK2N2 interactive modules.

Gene co-expression network analysis was carried out to investigate key genes related to EH in our patient cohort. We hypothesized that genes with high k-core values may be involved in hypertension. By calculating the Pearson’s correlation coefficient for each RNA pair, we constructed a co-expression network for 124 DE lncRNAs and 193 DE mRNAs with a k-core value ≥5 (Supplementary Figure 3A). A similar approach was used to correlate these 193 DE mRNAs with 178 DE circRNAs identified in hypertensive cases (Supplementary Figure 3B). This analysis revealed that 7 DE lncRNAs and 14 DE circRNAs were positively correlated with both WNT3 and CAMK2N2 (Figure 2A, 2B). Among the lncRNAs, LOC646616 and LAP3P2 showed the highest correlations (k-core value = 14), while for circRNAs, the highest correlations (k-core value = 20) were observed for hsa_circ_0039388 and hsa_circ_0038648. There was also a high correlation between WNT3 and CAMK2N2 (Supplementary Table 2).

### Establishment of a miRNA-based ceRNA regulatory network

Combined analysis of predicted interactions between the DE lncRNAs/circRNAs/mRNAs and previously annotated miRNAs was carried out to establish a ceRNA network. The results showed that 131 lncRNAs (98 upregulated and 33 downregulated), and 164 mRNAs (89 upregulated and 75 downregulated) bound to 1,475 miRNAs. In turn, 187 circRNAs (110 upregulated and 77 downregulated) and 159 mRNAs (84 upregulated and 75 downregulated) bound to 955 miRNAs. Subsequently, we constructed a miRNA-based sponge regulatory network to test the specific association of DE lncRNAs and circRNAs with WNT3 and CAMK2N2 (Supplementary Figure 4A), from which we derived a subnetwork that included only the most significant DE lncRNAs and circRNAs (Supplementary Figure 4B). This analysis showed that 5 and 12 miRNAs, respectively, could potentially mediate LOC646616/WNT3 and LOC646616/CAMK2N2 interactions (Figure 2C, 2D). Interestingly, we found that the LOC646616/miR-637 pair interacted with both WNT3 and CAMK2N2. In addition, it was predicted that 4 and 3 miRNAs, respectively, could mediate hsa_circ_0038648/WNT3 and hsa_circ_0038648/CAMK2N2 interactions (Supplementary Figure 4C, 4D). Expression data (in units of FPKM) for the most significant DE lncRNAs and circRNAs are shown in Supplementary Table 3.

### Validation of differentially expressed lncRNAs and circRNAs

**Figure 3:**
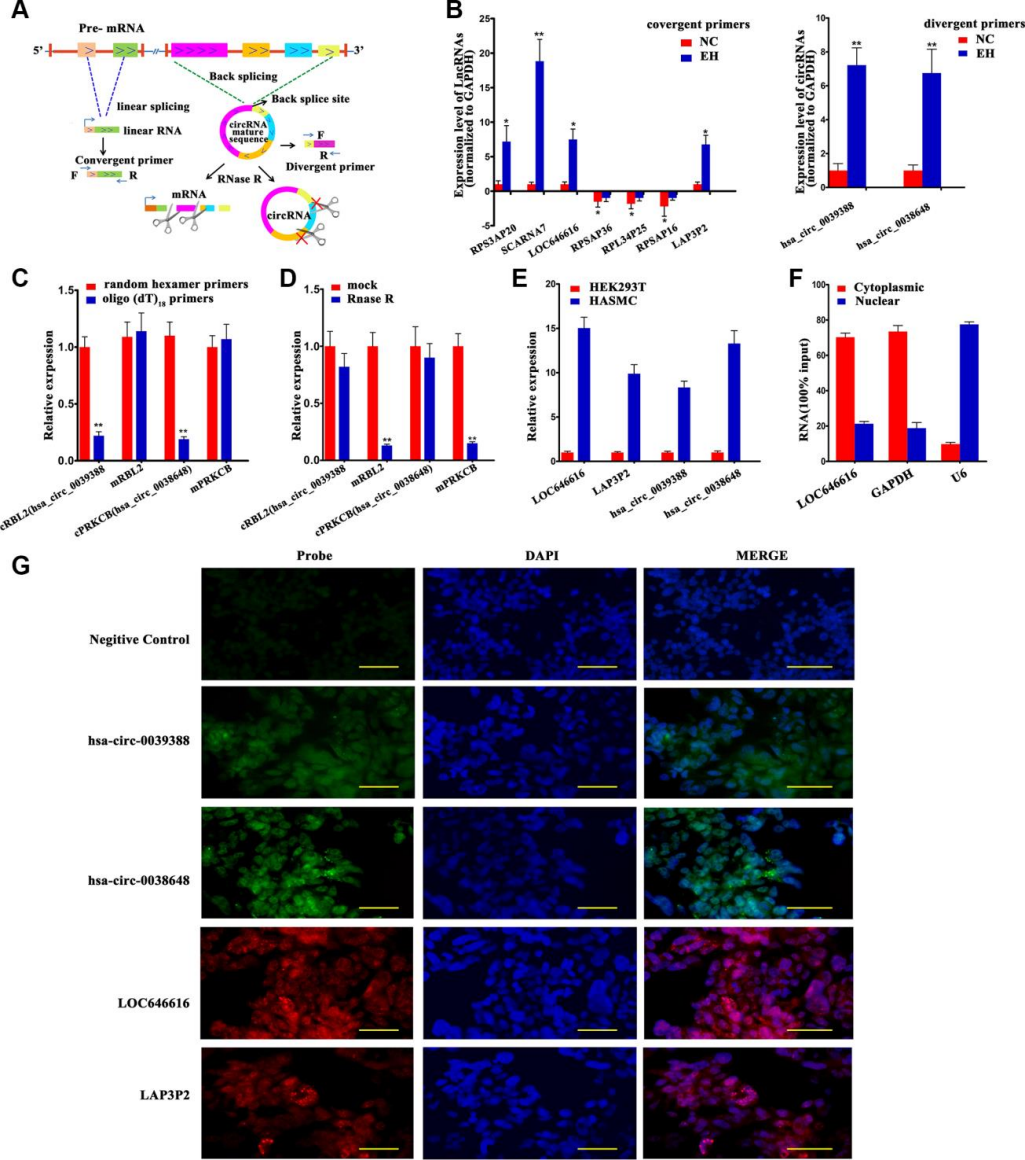
**Validation of DE lncRNAs and DE circNAs.** (**A**) Schematic diagram of validation patterns for DE lncRNAs and circRNAs. All samples were sequenced in the same batch. The screening criteria were log_2_ FC>1 or <-1, FDR<0.05. (**B**) qRT-PCR expression analysis of 7 DE lncRNAs (left panel) and 2 DE circRNAs (right panel) in PBMNCs from 60 hypertensive subjects and 60 normotensive controls. (**C**) qRT-PCR analysis of relative RNA levels generated using random hexamer or oligo (dT)_18_ primers in reverse transcription experiments. Data are normalized to those generated using random hexamer primers. (**D**) qRT-PCR analysis of 2 DE circRNAs and their linear host transcripts after treatment with RNase R. (**E**) Expression of LOC646616, LAP3P2, hsa_circ_0039388, and hsa_circ_0038648 detected by qRT-PCR in HASMCs and HEK293T cells. (**F**) Cytoplasmic and nuclear expression of LOC646616 in HASMCs measured by qRT-PCR. GAPDH served as cytoplasmic control, whereas U6 served as nuclear control. (**G**) RNA fluorescence in situ hybridization (FISH) analysis indicating predominantly cytoplasmic distribution for LOC646616 and hsa_circ_0038648 in HASMCs (red fluorescence indicates LAP3P2 and LOC646616 expression; green fluorescence indicates hsa_circ_0038648 and hsa_circ_0039388 expression). Scale bar = 20 μm. Data are presented as the mean ± SD. n = 3 biologically independent samples. *P<0.05, **P<0.01.

We selected 7 DE lncRNAs and 2 DE circRNAs (both exonic) for validation in 60 hypertensive and 60 normotensive Kazakh individuals (Figure 3A and Supplementary Table 4). The results of qRT-PCR assays for both lncRNAs (Figure 3B, left panel) and circRNAs (Figure 3B, right panel) were consistent with those from high-throughput RNA-Seq experiments (Supplementary Table 5). For circRNAs, we designed specific PCR primers verified through Sanger sequencing and agarose gel electrophoresis (Supplementary Figures 5 and 6). We characterized two DE circRNAs, termed cRBL2 (hsa_circ_0039388) and cPRKCB (hsa_circ_0038648), derived respectively from the RBL2 and PRKCB gene loci. To confirm the circular structure of these circRNAs, random hexamer or oligo (dT)_18_ primers were utilized for reverse transcription assays. When oligo (dT)_18_ primers were used, the relative expression of both cRBL2 and cPRKCB was clearly lower, while that of mRBL2 and mPRKCB remain unchanged (Figure 3C). This finding proved that cRBL2 and cPRKCB had no poly-A tail. Moreover, these circRNAs were more resistant to RNase R digestion than their linear host transcripts, suggesting that they were true circRNAs (Figure 3D). Next, we investigated the expression of LOC646616, LAP3P2, hsa_circ_0039388, and hsa_circ_0038648 in human aortic smooth muscle cells (HASMCs) and in Human embryonic kidney 293T (HEK293T) cells using qRT-PCR. Results showed high expression of all these transcripts in HASMCs (Figure 3E). To verify the subcellular localization of the above ncRNAs, we employed qRT-PCR. This analysis demonstrated that LOC646616 was mainly located in the cytoplasm (Figure 3F). Consistent with qRT-PCR results, single-molecule RNA-FISH analysis revealed a relatively high expression and a predominantly cytoplasmic distribution for both LOC646616 and hsa_circ_0038648 in HASMCs (Figure 3G).

### LOC646616 and hsa_circ_0038648 are independent risk factors for essential hypertension

**Figure 4:**
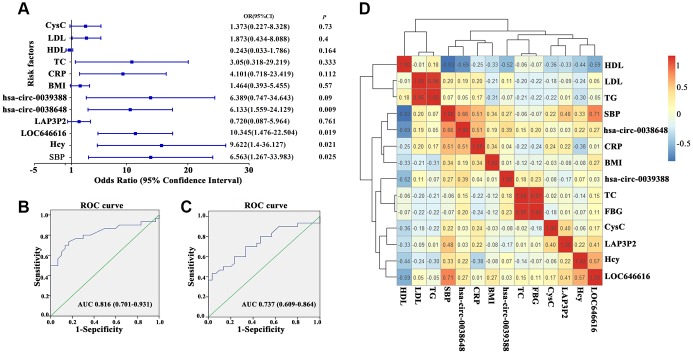
**Clinical correlations between DE ncRNAs and hypertension.** (**A**) Forest plot depicting logistic regression analysis results for the association between DE lncRNAs and circRNAs and general clinical data in hypertensive subjects. (**B**, **C**) Receiver operating characteristic (ROC) analysis of the relationship between LOC646616 and hsa_circ_0038648 expression and diagnosis of hypertension. (**D**) Heat map representation of a pairwise Spearman's correlation matrix for selected DE ncRNAs in hypertensive subjects (red and blue indicate positive and negative correlation, respectively; an r value of 1 or -1 indicates perfect correlation).

Logistic regression analysis conducted on our EH and control cohorts showed that LOC646616, hsa_circ_0038648, systolic blood pressure (SBP), and homocysteine (Hcy) levels were all independent risk factors for EH in Kazakh individuals (Figure 4A). To further investigate the relationship between LOC646616 and hsa_circ_0038648 expression and other clinical indicators, we performed multiple linear regression analysis. Results showed that SBP, Hcy, and Cystatin C (Cys C) were independent factors affecting the expression of LOC646616, whereas SBP and high-density lipoprotein (HDL) were independent factors affecting the expression of hsa_circ_0038648 (Supplementary Table 6).

On receiver operating characteristic (ROC) analysis, both LOC646616 and hsa_circ_0038648 showed significant association with diagnosis of hypertension. For LOC646616, the area under the curve (AUC) was 0.816 (CI 0.701-0.931, *P*<0.05), and sensitivity and specificity at the highest Youden index were 70.0% and 86.7%, respectively (Figure 4B). Meanwhile, for hsa_circ_0038648, the AUC was 0.737 (CI 0.609-0.864, *P*<0.05), and sensitivity and specificity (highest Youden index) were 70.0% and 66.7%, respectively (Figure 4C). These results indicated that assessment of LOC646616 and hsa_circ_0038648 expression has potential value in the clinical diagnosis of EH in the Kazakh population.

We also conducted correlation analysis between LOC646616, LAP3P2, hsa_circ_0039388, and hsa_circ_0038648 and general clinical data in the hypertension group. Utilizing the counts for each bin, we calculated the pairwise Spearman’s correlation coefficients for each pair of corrections to create a correlation coefficient matrix. A heat map of this matrix is shown in Figure 4D.

### LOC646616 inhibits miR-637 by direct binding

**Figure 5:**
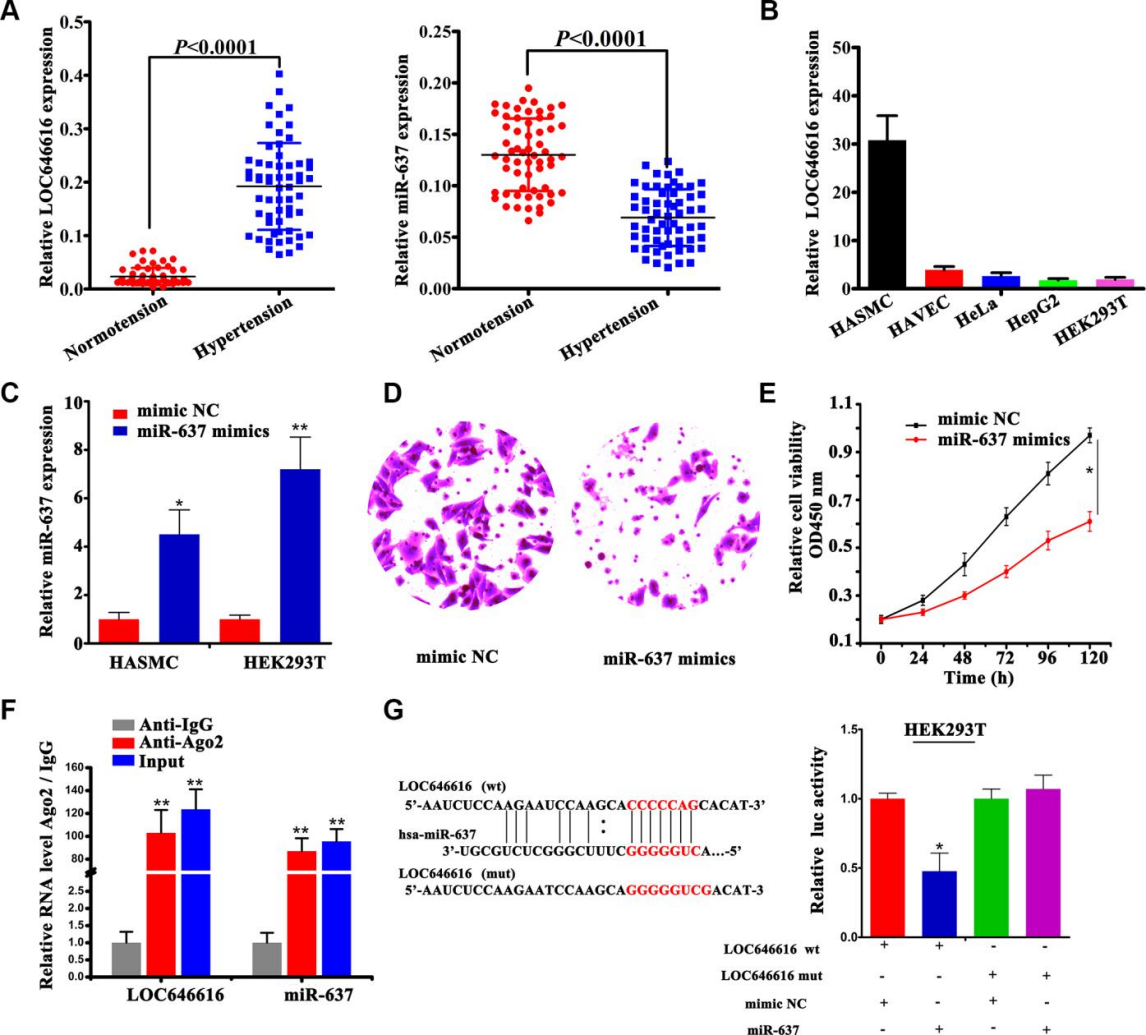
**Interaction between LOC646616 and miR-637.** (**A**) qRT-PCR analysis of relative LOC646616 and miR-637 expression in PBMNCs from hypertensive subjects and normotensive controls. (**B**) qRT-PCR analysis of relative LOC646616 expression in 5 cell lines. GAPDH was used for normalization. (**C**) qRT-PCR analysis of miR-637 expression in HASMCs and HEK293T cells transfected with miR-637 mimics or mimic NC. (**D**) Transwell invasion assay results from HASMCs transfected with miR-637 or mimic NC. (**E**) Results of CCK-8 viability assays in HASMCs with and without enforced miR-637 overexpression. (**F**) RIP assay results showing co-precipitation of LOC646616 and miR-637 by an Ago2 antibody in HASMCs. Verification of RIP products by qRT-PCR is also shown. (**G**) Bioinformatics evidence of the interaction between miR-637 and LOC646616, schematic diagram of the mutant LOC646616 luciferase reporter sequence, and results of luciferase activity assays in HEK293T cells co-expressing miR-637 mimics or mimic NC and reporter plasmids containing wild type (wt) or mutant (mut) LOC646616 sequences. Data are presented as the mean ± SD. *P<0.05, **P<0.01.

Our analysis showed that LOC646616 is significantly upregulated, while miR-637 expression is instead reduced in hypertensive subjects (Figure 5A). Since we predicted that LOC646616 might act as a ceRNA to regulate the expression of both WNT3 and CAMK2N2 through competition for miR-637, we conducted cellular expression studies on these non-coding RNAs to verify their interaction in situ. LOC646616 expression was examined by qRT-PCR in 5 human cell lines derived from different tissues. Results showed prominent expression of LOC646616 in HASMCs and weak expression in human arterial vascular endothelial cells (HAVECs) (Figure 5B). In addition, we detected the expression of miR-637 in HASMCs and HEK293T cells transfected with miR-637 mimics or negative control miRNA (mimic NC) (Figure 5C), and examined whether miR-637 overexpression affected invasive and proliferation potential in these cell lines. Transwell Matrigel invasion assays showed that miR-637 mimics transfection impaired invasive ability (Figure 5D) and reduced proliferative activity (Figure 5E) in HASMCs. To confirm the interaction between LOC646616 and miR-637, we performed RNA immunoprecipitation (RIP) and dual-luciferase reporter assays. RIP results revealed that LOC646616 and miR-637 were co-immunoprecipitated by an Ago2 antibody in HASMCs, indicating that both ncRNAs were in the same RNA-induced silencing complex (RISC). Subsequently, we confirmed through qRT-PCR that the precipitated products were indeed LOC646616 and miR-637 (Figure 5F). Considering the effect of miR-637 transfection, we conducted dual-luciferase reporter assays on HEK293T cells co-transfected with miR-637 mimics or mimic NC and luciferase constructs containing wild-type LOC646616 (Luc-LOC646616-wt) or a mutated transcript form (Luc-LOC646616-mut) (Figure 5G, left panel). Results showed that miR-637 expression suppressed luciferase activity driven by LOC646616-wt, and it had a much lower effect on its mutated form (Figure 5G, right panel). These results revealed that LOC646616 inhibits the expression of miR-637 by direct binding.

### miR-637 is a negative regulator of WNT3 expression

**Figure 6:**
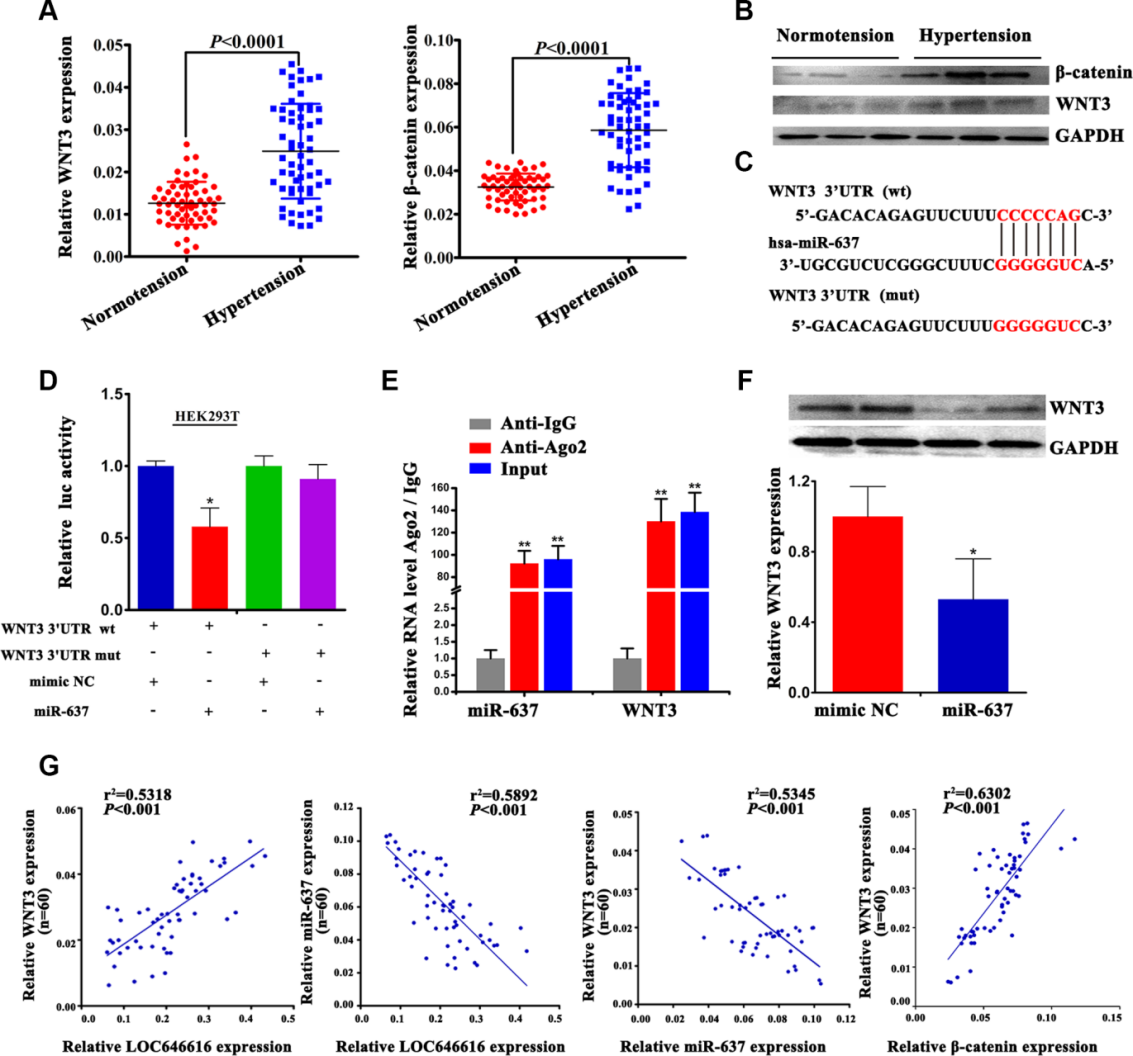
**WNT3 interacts directly with miR-637.** (**A**) qRT-PCR analysis of relative WNT3 and β-catenin expression in PBMNCs of hypertensive subjects and normotensive controls. (**B**) Western blot analysis of WNT3 and β-catenin levels in plasma of hypertensive subjects and normotensive controls. (**C**) Bioinformatics evidence of the interaction between miR-637 and the 3′-UTR of WNT3. Bottom: schematic diagram of the mutations in the WNT3 sequence used to create the mutant luciferase reporter construct. (**D**) Luciferase activity assay in HEK293T cells co-transfected with miR-637 mimics or mimic NC and luciferase report plasmids containing wild type (wt) or mutant (mut) WNT3 3′ UTR and. (**E**) RIP assay results showing co-precipitation of miR-637 and WNT3 mRNA complexes by an Ago2 antibody in HASMCs. Validation data obtained by qRT-PCR are also shown. (**F**) Western blot analysis of WNT3 levels in HASMCs transfected with miR-637 mimics or mimic NC. Expression data are normalized to GAPDH. (**G**) Spearman's correlation analysis of the relationship between LOC646616, WNT3, and miR-637 levels, and between WNT3 and β-catenin levels in our hypertensive cohort. Data are presented as the mean ± SD. n = 3 biologically independent samples. *P<0.05, **P<0.01.

Using qRT-PCR and western blot, we verified that WNT3 and β-catenin expression was considerably increased in hypertensive subjects (Figure 6A, 6B). Based on results of our ceRNA network analysis, we performed dual-luciferase reporter assays in HEK293T cells to confirm the interaction between miR-637 and WNT3. Results revealed that in the presence of miR-637 mimics the wild-type 3’-UTR of WNT3 exhibited a low translation level compared to the mutant 3’-UTR (Figure 6C, 6D). Furthermore, combined RIP-PCR assays showed that an Ago2 antibody was able to co-precipitate miR-637/WNT3 complexes in HASMCs (Figure 6E). To further verify that WNT3 is a direct target of miR-637, we transfected miR-637 mimics into HASMCs and examined WNT3 levels using western blot. We found that upregulation of miR-637 expression led to a significant decrease in WNT3 expression (Figure 6F), thus confirming that WNT3 protein levels are negatively regulated by miR-637. Finally, we performed Spearman’s correlation analysis on our hypertensive cohort and found a positive correlation between WNT3 and LOC646616 expression, whereas miR-637 expression was inversely correlated with both LOC646616 and WNT3 levels (Figure 6G). This analysis also showed that WNT3 levels were positively correlated with those of β-catenin. Taken together, these findings provide clear evidence that WNT3 is a direct target of miR-637 and suggest a critical role for miR-637 as a regulator of WNT3/β-catenin protein expression in EH.

### LOC646616 indirectly activates WNT3 by sponging miR-637

**Figure 7:**
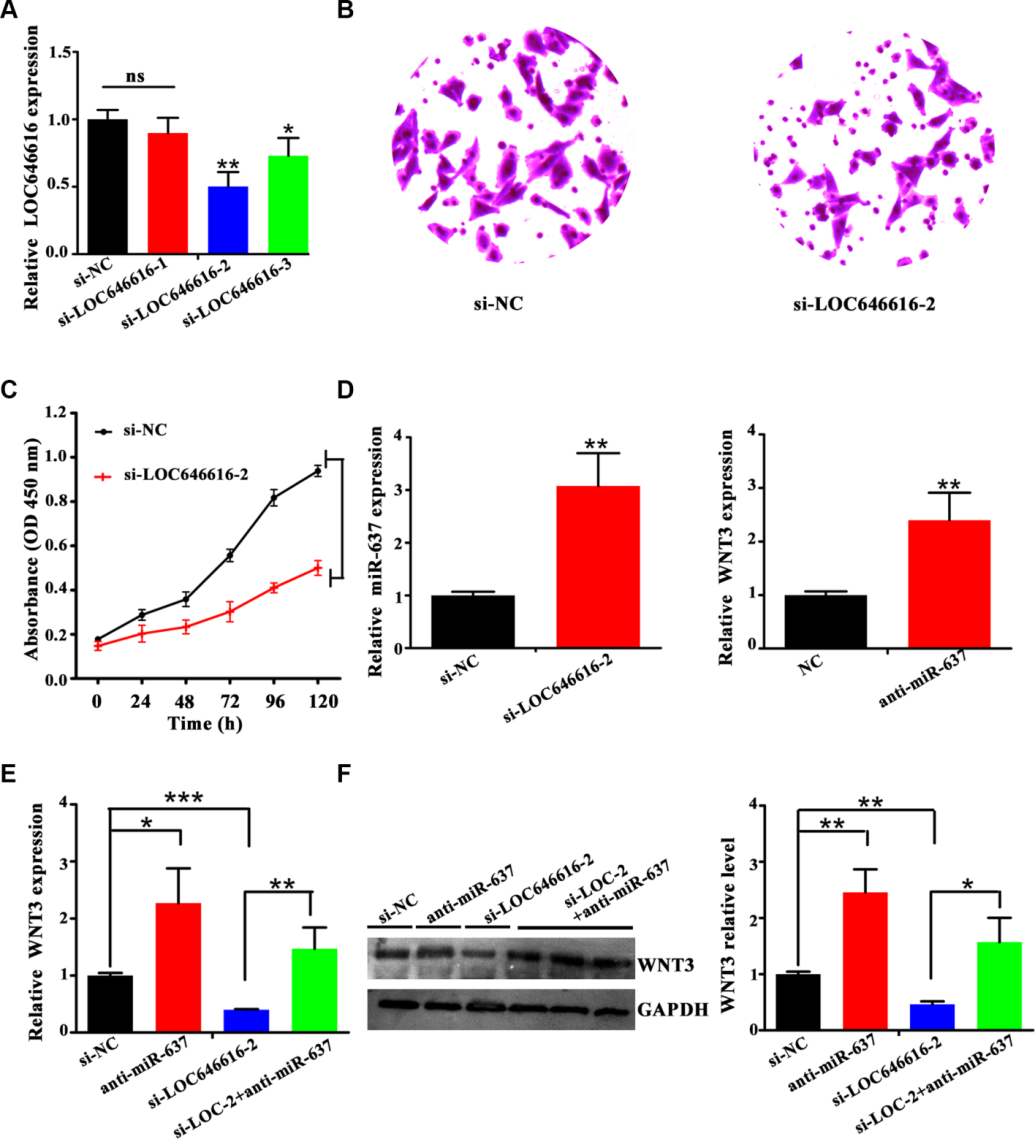
**LOC646616 increases WNT3 expression by sponging miR-637.** (**A**) qRT-PCR analysis of LOC646616 expression in HASMCs transfected with control siRNA (si-NC) or with 3 siRNA variants targeting LOC646616. (**B**, **C**) Results of Transwell invasion and viability (CCK-8) assays conducted in HASMCs transfected with si-LOC646616-2. (**D**) Left panel: relative miR-637 expression in HASMCs transfected with si-NC or si-LOC646616-2. Right panel: relative WNT3 mRNA expression in HASMCs transfected with NC or anti-miR-637. (**E**, **F**) WNT3 mRNA (left panel) and protein (middle and right panel) levels in HASMCs following knockdown of LOC646616 (si-LOC-2) with/without concurrent inhibition of miR-637. *P<0.05, **P<0.01, ***P<0.001.

To investigate the potential function of LOC646616 in HASMCs, we performed siRNA-mediated knockdown. Among the 3 probes tested, siRNA-LOC646616-2 (si-LOC646616-2) showed the highest knockdown efficiency (Figure 7A) and was therefore selected for use in subsequent experiments. Transwell Matrigel invasion assays and CCK-8 proliferation assays revealed that LOC646616 knockdown decreased both invasive capacity (Figure 7B) and proliferative ability (Figure 7C) in HASMCs. In line with the findings presented thus far, we found that when LOC646616 was silenced miR-637 expression increased significantly (Figure 7D, left panel). In turn, when HASMCs were transfected with miR-637 inhibitor (anti-miR-637), WNT3 expression increased significantly (Figure 7D, right panel). To verify that LOC646616 interacts with miR-637 to activate the expression of WNT3, we measured WNT3 mRNA and protein levels in HASMCs transfected with siRNAs targeting the corresponding ncRNAs. As shown in Figure 7E and 7F, WNT3 expression was significantly reduced by LOC646616 knockdown, and significantly increased by miR-637 inhibition. Confirming opposite regulatory roles for these ncRNAs on WNT3 expression, further expression assays showed that concurrent inhibition of miR-637 counteracted the inhibitory effect of LOC646616 silencing on WNT3 expression (Figure 7E and 7F). These results strongly suggest that LOC646616 increases WNT3 expression by sponging miR-637.

### miR-637 is a negative regulator of CAMK2N2 expression

Based on the results of our ceRNA network predicting a regulatory module composed of LOC646616-miR-637-CAMK2N2, we assessed CAMK2N2 expression and conducted correlation analyses for this ceRNA triplet. Both qRT-PCR and western blotting showed that CAMK2N2 expression was significantly higher in hypertensive subjects compared to normotensive controls (Supplementary Figure 7A, 7B). Meanwhile, bivariate correlation analysis demonstrated that CAMK2N2 was positively correlated with LOC646616 (Supplementary Figure 7C) and inversely correlated with miR-637 (Supplementary Figure 7D). These data strongly suggest that LOC646616 and miR-367 exert mutual regulation on both WNT3 and CAMK2N2, and indicate that LOC646616 upregulation inhibits miR-637 expression to activate the WNT3/β-catenin axis in hypertension.

## DISCUSSION

Using RNA-seq and bioinformatic analyses, our study identified a set of lncRNAs and circRNAs with differential expression in hypertensive individuals from the Kazakh population in Xinjiang, China. Based on RNA co-expression and miRNA-based regulatory network analyses, we demonstrate that LOC646616 (Homo sapiens transmembrane protein 183A pseudogene; TMEM183A), a 1,379-nucleotide-long lncRNA transcribed from human chromosome 5, is upregulated in EH patients and acts as a ceRNA to positively regulate WNT3 and CAMK2N2 expression by sponging miR-637. This novel finding sheds light on a potentially important mechanism leading to activation of the WNT/β-catenin signaling pathway in hypertension.

Although research on lncRNA functions in EH is still scarce, several lncRNAs have been shown to be upregulated by angiotensin-II, highlighting important contributions to vascular diseases, including hypertension [23]. Examples include AK098656, which promotes hypertension through regulation of vascular smooth muscle cells (VSMCs) functions [24], GAS5, which promotes vascular remodeling and is downregulated in hypertension [25], and the TUG1/miR-145-5p/FGF10 axis, which regulates proliferation and migration in VSMCs [26].

We detected 1,002 DE circRNAs in hypertensive subjects. Although numerous circRNAs have been shown to influence the occurrence and development of human diseases [27, 28], only a few have so far been linked to hypertension. For instance, recent studies showed that two circRNAs, hsa_circ_0005870 and hsa_circ_0037911, which are respectively upregulated and downregulated in hypertensive subjects, could serve as valuable diagnostic biomarkers [29, 30]. Interestingly, our ceRNA network showed that besides LOC646616, another lncRNA, namely LAP3P2, and two circRNAs, i.e. hsa_circ_0039388 and hsa_circ_0038648, were also highly co-expressed with both WNT3 and CAMK2N2 mRNAs in hypertensive subjects, while qRT-PCR showed effective expression of these four ncRNAs in cultured HASMCs. Although future validation experiments are warranted, these data would suggest that WNT3/β-catenin and CAMK2N2 (Ca^2+^/CAMKII) pathway activities are influenced by regulation imparted by multiple ncRNAs.

Using miRNA target prediction algorithms, we identified miR-637 as a putative binding partner of LOC646616 and predicted through further analysis a regulatory network conformed by LOC646616/miR-637/WNT3 with potential relevance in EH. After assessing that miR-637 was downregulated in hypertensive samples, we conducted miR-637 overexpression experiments that led to significant downregulation of its target WNT3 and impaired both motility and survival in HASMCs. On the other hand, the combined results of qRT-PCR and FISH-RNA assays showed that LOC646616 was prominently expressed in HASMCs, rather than HAVECs, and was mainly localized in the cytoplasm, which is consistent with a potential function in mRNA translation. Subsequently, luciferase reporter assays in HASMCs demonstrated that LOC646616 interacted directly with miR-637, while RIP assays showed that they could be co-detected in association with the RISC complex. The WNT/β-catenin signaling pathway is an important mediator of cardiovascular development and has shown to influence atherosclerosis by regulating inflammation, vascular calcification, and the proliferation of different cells within vascular tissues [31, 32]. Previous reports demonstrated that the WNT/β-catenin pathway is activated in hypertension [33], while miR-637 has been linked cardiovascular disease, acute ischemic stroke, and different cancers [34–36]. The present data adds to our understanding of the regulation of WNT/β-catenin signaling in hypertension, highlighting a novel mechanism by which LOC646616 binds miR-637 to relieve its inhibition on WNT3 expression.

**Figure 8:**
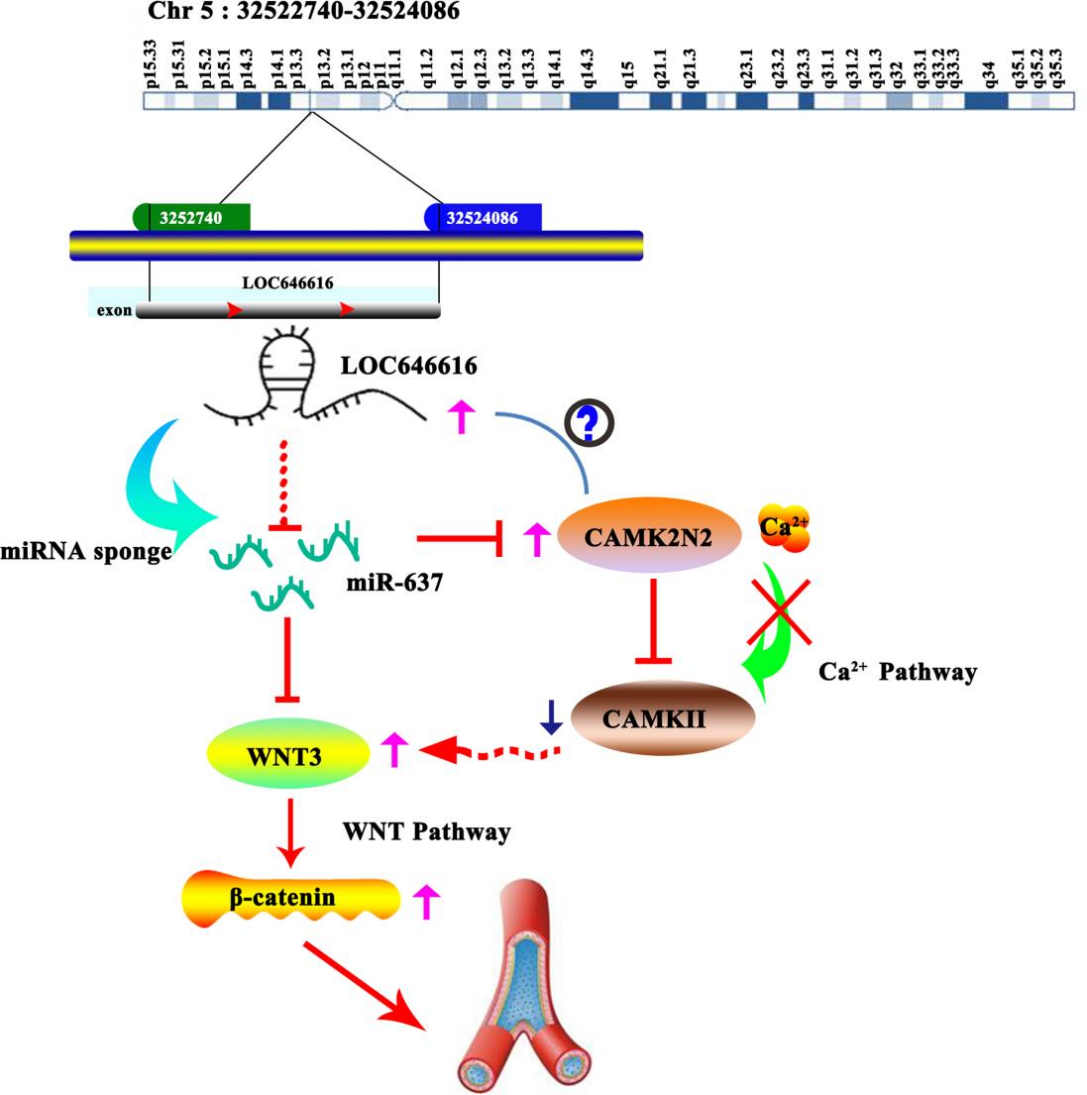
**Schematic representation of the LOC646616/miR-637/WNT3 ceRNA network in EH.** LOC646616 indirectly promotes WNT3 mRNA translation by sponging miR-637, leading to WNT3/β-catenin pathway activation.

Although validation in cellular models is still required, combined data from our expression and bioinformatics analyses suggest that the LOC646616/miR-637 pair also controls CAMK2N2 protein expression. CAMK2N2 is a specific inhibitor of CAMKII through binding to the kinase’s catalytic domain. It has been proved that enhanced contractile responses mediated by alamandine in cardiomyocytes from hypertensive rats occur through NO-dependent activation of CAMKII [37]. Research also showed that CAMKII activation (e.g. by non-canonical WNT/Ca^2+^ signaling) can indirectly silence transcription mediated by the canonical WNT/β-catenin pathway by stimulating TAK1-NLK MAPK activities [18]. Therefore, we propose that by releasing miR-637-mediated inhibition of both WNT3 and CAMK2N2 expression, LOC646616 upregulation in hypertension promotes WNT/β-catenin pathway activation both directly, through enhanced WNT3 expression, and indirectly, by preventing CAMKII-mediated inhibition (Figure 8).

Importantly, results of our logistic regression analysis indicated that LOC646616 and hsa_circ_0038648 were independent risk factors for EH of Kazakh subjects, and both were notably correlated with SBP levels. In turn, our ROC analysis implied that these ncRNAs may serve as valuable biomarkers for hypertension. However, more extensive work is needed to define if changes in LOC646616 and hsa_circ_0038648 expression are indeed causative or are rather a consequence of hypertension. To this end, the use of well-validated, standardized protocols that produce reliable and repeatable results will help translate basic research findings into clinical practice, with a potentially significant impact on public health [38].

In summary, our findings unmasked a set of potential ceRNA biomarkers of hypertension in Kazakh individuals that may improve diagnostic/prognostic accuracy for this condition. Still, future work is needed to verify whether our results extend to other populations. Importantly, our research also highlights a potentially critical role for the lncRNA LOC646616 in the activation of the WNT/β-catenin pathway by relieving the negative regulation exerted by miR-637 on both WNT3 and CAMK2N2 expression in human vascular smooth muscle cells during hypertension. Given the mounting evidence linking various ncRNA species to the onset and progression of cardiovascular diseases, in-depth study of their expression patterns and biological functions should improve risk prediction efforts and identify new therapeutic targets to develop individualized treatments.

## MATERIALS AND METHODS

### Study population

The study was approved by the ethics committee of the First Affiliated Hospital of Shihezi University School of Medicine in China and conducted in accordance with current (2013) guidelines of the Helsinki Declaration of ethical principles in human research. All the participants were Kazakhs living in or near the Xinjiang Uygur Autonomous Region and were recruited after informed consent at the First Affiliated Hospital of Shihezi University between March 2016 and July 2017. Diagnostic criteria for hypertension were based on the 2018 guidelines for hypertension in China [39]. Molecular studies were first performed on 3 EH patients (2 females and 1 male) and 5 normotensive individuals (2 females and 3 males). Another 60 EH cases, as well as 60 sex- and age- (±3 years) matched healthy controls, were recruited for further validation and analysis. In total, two independent cohorts involving 63 EH subjects and 65 healthy controls were defined.

### RNA extraction, sequencing, and quality control

Following collection of 4 ml of venous blood in EDTA-coated anticoagulation tubes, PBMNCs were isolated by density gradient centrifugation for 30 min at 4000 × g using Lymphocyte Separation Medium (cat No. P8610, Solarbio, Shanghai, China). Total RNA was extracted using Trizol reagent (Invitrogen, CA, USA) and quantified on an Agilent 2200 TapeStation (Agilent, CA, USA). RNA samples with a RIN>7 were used for cDNA library construction using Illumina TruSeq RNA Library Prep Kit v2, according to the protocol provided by the manufacturer (Illumina, CA, USA). Sequencing depths >10 Gb were obtained for each sample. High throughput sequencing was completed by Shanghai Novel Bioinformatics Co., Ltd. We used Fast-QC (http://www.bioinformatics.babraham.ac.uk/projects/fastqc/) software to perform an overall assessment of the quality of the sequencing data, including distributions of base mass and mass value position, GC content, analysis of PCR duplicates, and k-mer frequency. The overall workflow is show in Supplementary Figure 8A, 8B.

### Differential expression, GO, and KEGG pathway analyses

The screening criteria to consider a given circRNA, lncRNA, or mRNA as DE were log_2_ FC>1 or <-1 and FDR <0.05. Functional and pathway enrichment analyses of DE transcripts were conducted by accessing the GO (http://geneontology.org/) and KEGG (http://www.genome.jp/kegg/) databases. *P*<0.05 indicated significance for both GO terms and KEGG pathways.

### Competing endogenous RNA network construction

A ceRNA network describing lncRNA/circRNA–miRNA–mRNA interactions was constructed based on the hypothesis that lncRNAs/circRNAs increase or decrease the activity of mRNAs by acting as miRNA sponges [40]. The process consisted of three steps: first, we predicted putative miRNA targets for the identified DE lncRNAs, circRNAs, and mRNAs using miRanda (pairing score > 150; MFE <-20 kcal/mol) and RNAhybrid (MFE <-25 kcal/mol); second, we selected the final target list from the intersection results of the two prediction methods; third, we correlated this list with our DE ncRNA data to identify lncRNA/circRNA-miRNA-mRNA networks (pick up-/-up or down-/-down relationship entries). *P* <0.05 was considered significant.

### Co-expression network analysis

The co-expression network analysis was based on calculation of the Pearson’s correlation coefficient between coding and noncoding genes according to their expression levels. Genes with *P* <0.05 and FDR <0.05 were retained for further analysis. We obtained k-core scores to identify core regulatory genes with the highest networking degrees in the networks [41].

### Cell culture

HEK293T, HAVECs, and HASMCs cells were purchased from Enzyme Biotechnology (Shanghai, China). HepG2 and HeLa cells were purchased from ATCC (Manassas, VA, USA). HAVECs were cultured in Endothelial Cell Medium (cat No.1001, ScienCell research laboratory, USA). The other cell lines were cultured in DMEM/high-glucose medium (cat. 1859228, Gibco, CA, USA) at 37 °C with 5% CO_2_. Culture media were supplemented with 10% fetal bovine serum (FBS; cat No. 04-001-1A/B/C, Biological Industries, Kibbutz Beit Haemek, Israel), and 1% penicillin-streptomycin solution (cat No. P1400, Solarbio, Beijing, China).

### Transwell invasion assay

Cell invasion was evaluated in 24-well plates fitted with 8-μm pore Transwell polycarbonate membrane inserts (Corning, NY, USA) coated with 35 μl Matrigel. Briefly, after 24 h of transient transfection with miR-637 mimics or mimic NC, 200 μl of cell suspension (1×10^5^ cells) was added to the upper chamber, while the lower chamber was filled with DMEM (KeyGen, Nanjing, China) containing 10% FBS. After 36-h incubation, the membranes were fixed and stained with Giemsa. Invading cells were counted in five fields of view per well under light microscopy. Each condition was assessed in triplicate.

### Cell proliferation assay

Cell proliferation was evaluated with the Cell Counting Kit-8 assay (CCK-8; cat. YB-0050, Yiyuan, Guangzhou, China) according to the manufacturer’s instructions. Briefly, cells were seeded at 1×10^3^ cells/well in 96-well plates. After 24, 48, 72, 96, or 120 h of incubation, 10 μl CCK-8 reagent was added into each well, followed by incubation for 2 h at 37 °C. Subsequently, absorbance was measured at 450 nm using a microplate reader (Power WaveXS2; Biotek, Winooski, VT, USA). Each experiment was conducted in triplicate.

### RNA fluorescence in situ hybridization (RNA-FISH)

The subcellular localization of LOC646616, LAP3P2, hsa_circ_0038648, and hsa_circ_0039388 was detected in HASMCs using a FISH probe kit (BOSTER, Wuhan, China). In brief, HASMCs were fixed in 4% paraformaldehyde for 20 min at room temperature, prehybridized with a hybridization solution, and incubated with LOC646616 (cat. MK10530-h), LAP3P2 (cat. MK10529-h), hsa-circ-0038648 (cat. MK10528) and hsa-circ-0039388 (cat. MK10528) probes at 37 °C for 30 min. The sections were stained with anti-digoxin rhodamine conjugate (Boster) at 37 °C for 2 h. Then, the sections were stained with SABC-FITC or SABC-cy3 at 37 °C for 30 min away from light. The sections were subsequently stained with 4′,6-diamidino-2-phenylindole (DAPI, Beyotime, China) for nuclear visualization. The LOC646616 probe sequences were 5'- Cy3-ATATTTGGCTATTGCTGGC CTCCTATATCTGTCCTGAGGA-3’; 5'- Cy3-TACGGCATCATTGAGTGAGACT GGTGTTTCAAGATTCCCC-3’; 5'- Cy3-TAGTAATGTACATGCTCTTCAGGTT CTAGGGCTCCTGTTA-3’. The LAP3P2 probe sequences were 5'- Cy3- CTCTCT TCCGTGGAGGTGGATCCCTGTAGAGATGCTCAGG-3’; 5'- Cy3-ATTGTGTCT GCTGCAAATCTCAGTTTGCCCATTAATATTA-3’; 5'- Cy3-GACTCTCATAGA GTTCTTACTTCGTTTCAGTCAAGACAAT-3’. The hsa_circ_0039388 probe sequence was FITC-5’-AACTTGTGTTTGCTGCGGGGGTCCAGCCATCAAC ACTGGCTTTCTGAA-3’- FITC. The hsa_circ_0038648 probe sequence was FITC- 5’-CTGGAAGGACTTATCTTCTTTTGTGACATTTGCCTGTGAGTACTTCATGGCAAA-3’- FITC. Cell nuclei were stained with 4′, 6-diamidino-2-phenylindole (DAPI) for 5 min at room temperature. The lncRNAs were stained with SABC-cy3 (Red) and the circRNAs were stained with SABC-FITC (Green), and visualized under a fluorescence microscope (CKX53; Olympus, Tokyo, Japan).

### Quantitative real-time polymerase chain reaction (qRT-PCR)

Total RNA was extracted using TRIzol (Invitrogen, CA, USA). The purity and concentration of RNA samples were quantified using a NanoDrop ND-1000 device (NanoDrop Technologies/Thermo Scientific, Wilmington, DE, USA). Total RNA (2 μg) from each sample was reverse transcribed using a Hairpin-it qRT-PCR kit (GenePharma Co., Shanghai, China) according to the manufacturer’s instructions. LncRNAs and circRNAs levels were normalized to GAPDH mRNA. Isolation of miRNAs was performed using a miRNA Isolation kit (Thermo Fisher Scientific, Waltham, USA), and a Hairpin-it miRNA qPCR Quantitation kit (GenePharma Co.) was used to measure miRNA levels according to the manufacturer's instructions. Relative miRNA gene expression was normalized to U6. Reverse transcription reactions were conducted using convergent primers for mRNAs and lncRNAs, divergent primers for circRNAs, and stem-loop RT primers for miRNAs (GenePharma). Relative changes in gene expression were measured using the 2^−ΔΔCT^ method. Primer sequences are shown in Supplementary Table 7.

### Western blotting

Treated HASMCs and PBMNCs were lysed in RIPA buffer and protein content was determined using a BCA Protein Assay Kit (cat. P0011, Beyotime). Proteins were separated by 12% or 9% SDS-PAGE and transferred to PVDF membranes (Millipore, MA, USA). Primary antibodies against WNT3 (cat. GTX89319; Gene Tex, CA, USA), β-catenin (cat. GTX101435-S; Gene Tex), calcium/calmodulin-dependent protein kinase II inhibitor 2 (CAMK2N2, cat. TA322113S; Origene, MD, USA), and GAPDH (cat. TA-08; ZSGB-BIO, Beijing, China) were applied at 4 °C overnight. Subsequently, a horseradish peroxidase-conjugated secondary antibody (1:10000) was applied at room temperature for 1 h. The signals were detected with a SuperSignal West Femto Trial Kit (Thermo Fisher Scientific). Relative protein content was analyzed by ImageJ software and normalized to loading controls.

### miRNA mimics/ inhibitor transfection

Cells grown overnight were transfected over 48 h with miR-637 mimics/inhibitor or its negative control (both designed and synthesized by GenePharma, Shanghai, China) using Lipofectamine 2000 (Invitrogen, CA, USA) according to the manufacturer’s protocol.

### Dual-luciferase reporter assay

Luciferase reporters carrying wild-type (wt) or mutated (mut) miR-637 binding sites in the WNT3 3’UTR or LOC646616 sequences (designed and synthesized by GenePharma) were inserted into the pmirGLO dual-luciferase reporter vector. HEK293T cells were plated in 96-well plates and then co-transfected with 50 nmol/L of constructs along with miR-637 mimics or mimic NC. After 48 h, luciferase activity was detected using a dual-luciferase reporter assay system (Promega, Madison, WI, USA) according to the manufacturer’s instructions and normalized to Renilla activity. Experiments were performed in triplicate.

### RIP

The RIP assay was carried out using a Magna RIP RNA Binding Protein Immunoprecipitation Kit (Millipore, Billerica, MA, USA) according to the manufacturer’s instructions. Lysed cells were incubated in RIP wash buffer, which contained magnetic beads bound to an antibody recognizing the human Ago2 protein or to immunoglobulin G (IgG). Extraction of RNA after digestion with proteinase K. Immunoprecipitated RNA was detected by qRT-PCR. All laboratory supplies are guaranteed to be free of DNase and RNase, and three sets of repetitions per group.

### Transient transfection with siRNAs

HASMCs (5 × 10^4^ cells/well) were seeded in 6-well plates and cell confluence reached 70-90% within 24 h. Then transfected with siRNAs (100 pmol) targeting LOC646616 (si-LOC646616) or with corresponding negative siRNA controls (si-NC) using Lipofectamine reagent (5 μL). Incubate in CO_2_ incubator at 37 °C for 24-48h, after which RNA expression was examined using qRT-PCR. All siRNAs were purchased from GenePharma, and three biological replicates were assayed in each experiment. The relevant siRNA sequences are shown in Supplementary Table 8.

### Statistical analysis

All the data were checked for normality and homogeneity of variance, and are expressed as the mean ± SD. Student’s *t*-test was used to compare differences between the means of two groups. Non-parametric Mann-Whitney test was used to compare non-normal distributions. Correlation analysis of the two sets of data asked using Spearman’s correlation analysis. Data analysis and graphs were completed using the NovelBrain^®^ cloud computing platform. Statistical analyses were performed with GraphPad Prism 5.01 (GraphPad, San Diego, CA, USA) and SPSS 17.0 for Windows (SPSS Inc, Chicago, IL, USA). Significance was defined as *P* < 0.05.

## Supplementary Material

Supplementary Figures

Supplementary Tables

## References

[1] Kearney PM, Whelton M, Reynolds K, Muntner P, Whelton PK, He J. Global burden of hypertension: analysis of worldwide data. Lancet. 2005; 365:217–23. 10.1016/S0140-6736(05)17741-1 15652604

[2] Liu F, Adi D, Xie X, Li XM, Fu ZY, Shan CF, Huang Y, Chen BD, Gai MT, Gao XM, Ma YT, Yang YN. Prevalence of Isolated Diastolic Hypertension and Associated Risk Factors among Different Ethnicity Groups in Xinjiang, China. PLoS One. 2015; 10:e0145325. 10.1371/journal.pone.0145325 26694755 PMC4690591

[3] Dominiczak AF, Munroe PB. Genome-wide association studies will unlock the genetic basis of hypertension: pro side of the argument. Hypertension. 2010; 56:1017–20. 10.1161/HYPERTENSIONAHA.110.156208 21060004

[4] Gupta RA, Shah N, Wang KC, Kim J, Horlings HM, Wong DJ, Tsai MC, Hung T, Argani P, Rinn JL, Wang Y, Brzoska P, Kong B, et al. Long non-coding RNA HOTAIR reprograms chromatin state to promote cancer metastasis. Nature. 2010; 464:1071–76. 10.1038/nature08975 20393566 PMC3049919

[5] Wu G, Cai J, Han Y, Chen J, Huang ZP, Chen C, Cai Y, Huang H, Yang Y, Liu Y, Xu Z, He D, Zhang X, et al. LincRNA-p21 regulates neointima formation, vascular smooth muscle cell proliferation, apoptosis, and atherosclerosis by enhancing p53 activity. Circulation. 2014; 130:1452–65. 10.1161/CIRCULATIONAHA.114.011675 25156994 PMC4244705

[6] Morán I, Akerman I, van de Bunt M, Xie R, Benazra M, Nammo T, Arnes L, Nakić N, García-Hurtado J, Rodríguez-Seguí S, Pasquali L, Sauty-Colace C, Beucher A, et al. Human β cell transcriptome analysis uncovers lncRNAs that are tissue-specific, dynamically regulated, and abnormally expressed in type 2 diabetes. Cell Metab. 2012; 16:435–48. 10.1016/j.cmet.2012.08.010 23040067 PMC3475176

[7] Shi T, Gao G, Cao Y. Long Noncoding RNAs as Novel Biomarkers Have a Promising Future in Cancer Diagnostics. Dis Markers. 2016; 2016:9085195. 10.1155/2016/9085195 27143813 PMC4842029

[8] Qu S, Yang X, Li X, Wang J, Gao Y, Shang R, Sun W, Dou K, Li H. Circular RNA: A new star of noncoding RNAs. Cancer Lett. 2015; 365:141–48. 10.1016/j.canlet.2015.06.003 26052092

[9] Salmena L, Poliseno L, Tay Y, Kats L, Pandolfi PP. A ceRNA hypothesis: the Rosetta Stone of a hidden RNA language? Cell. 2011; 146:353–58. 10.1016/j.cell.2011.07.014 21802130 PMC3235919

[10] Tay Y, Rinn J, Pandolfi PP. The multilayered complexity of ceRNA crosstalk and competition. Nature. 2014; 505:344–52. 10.1038/nature12986 24429633 PMC4113481

[11] Li Y, Huo C, Lin X, Xu J. Computational Identification of Cross-Talking ceRNAs. Adv Exp Med Biol. 2018; 1094:97–108. 10.1007/978-981-13-0719-5_10 30191491

[12] Pahnke A, Conant G, Huyer LD, Zhao Y, Feric N, Radisic M. The role of Wnt regulation in heart development, cardiac repair and disease: A tissue engineering perspective. Biochem Biophys Res Commun. 2016; 473:698–703. 10.1016/j.bbrc.2015.11.060 26626076 PMC4854783

[13] Abou Ziki MD, Mani A. Wnt signaling, a novel pathway regulating blood pressure? State of the art review. Atherosclerosis. 2017; 262:171–78. 10.1016/j.atherosclerosis.2017.05.001 28522145 PMC5508596

[14] Tortelote GG, Hernández-Hernández JM, Quaresma AJ, Nickerson JA, Imbalzano AN, Rivera-Pérez JA. Wnt3 function in the epiblast is required for the maintenance but not the initiation of gastrulation in mice. Dev Biol. 2013; 374:164–73. 10.1016/j.ydbio.2012.10.013 23085236 PMC3551248

[15] Kim M, Lee HC, Tsedensodnom O, Hartley R, Lim YS, Yu E, Merle P, Wands JR. Functional interaction between Wnt3 and Frizzled-7 leads to activation of the Wnt/β-catenin signaling pathway in hepatocellular carcinoma cells. J Hepatol. 2008; 48:780–91. 10.1016/j.jhep.2007.12.020 18313787 PMC2390890

[16] Prasad AM, Morgan DA, Nuno DW, Ketsawatsomkron P, Bair TB, Venema AN, Dibbern ME, Kutschke WJ, Weiss RM, Lamping KG, Chapleau MW, Sigmund CD, Rahmouni K, Grumbach IM. Calcium/calmodulin-dependent kinase II inhibition in smooth muscle reduces angiotensin II-induced hypertension by controlling aortic remodeling and baroreceptor function. J Am Heart Assoc. 2015; 4:e001949. 10.1161/JAHA.115.001949 26077587 PMC4599535

[17] Zhang M, Hagenmueller M, Riffel JH, Kreusser MM, Bernhold E, Fan J, Katus HA, Backs J, Hardt SE. Calcium/calmodulin-dependent protein kinase II couples Wnt signaling with histone deacetylase 4 and mediates dishevelled-induced cardiomyopathy. Hypertension. 2015; 65:335–44. 10.1161/HYPERTENSIONAHA.114.04467 25489064

[18] Ishitani T, Kishida S, Hyodo-Miura J, Ueno N, Yasuda J, Waterman M, Shibuya H, Moon RT, Ninomiya-Tsuji J, Matsumoto K. The TAK1-NLK mitogen-activated protein kinase cascade functions in the Wnt-5a/Ca(2+) pathway to antagonize Wnt/beta-catenin signaling. Mol Cell Biol. 2003; 23:131–39. 10.1128/MCB.23.1.131-139.2003 12482967 PMC140665

[19] Zarkou V, Galaras A, Giakountis A, Hatzis P. Crosstalk mechanisms between the WNT signaling pathway and long non-coding RNAs. Noncoding RNA Res. 2018; 3:42–53. 10.1016/j.ncrna.2018.04.001 30159439 PMC6096407

[20] You BH, Yoon JH, Kang H, Lee EK, Lee SK, Nam JW. HERES, a lncRNA that regulates canonical and noncanonical Wnt signaling pathways via interaction with EZH2. Proc Natl Acad Sci USA. 2019; 116:24620–29. 10.1073/pnas.1912126116 31732666 PMC6900598

[21] Guan H, Zhu T, Wu S, Liu S, Liu B, Wu J, Cai J, Zhu X, Zhang X, Zeng M, Li J, Song E, Li M. Long noncoding RNA LINC00673-v4 promotes aggressiveness of lung adenocarcinoma via activating WNT/β-catenin signaling. Proc Natl Acad Sci USA. 2019; 116:14019–28. 10.1073/pnas.1900997116 31235588 PMC6628810

[22] Wu G, Jose PA, Zeng C. Noncoding RNAs in the Regulatory Network of Hypertension. Hypertension. 2018; 72:1047–59. 10.1161/HYPERTENSIONAHA.118.11126 30354825 PMC6208146

[23] Leung A, Trac C, Jin W, Lanting L, Akbany A, Sætrom P, Schones DE, Natarajan R. Novel long noncoding RNAs are regulated by angiotensin II in vascular smooth muscle cells. Circ Res. 2013; 113:266–78. 10.1161/CIRCRESAHA.112.300849 23697773 PMC3763837

[24] Jin L, Lin X, Yang L, Fan X, Wang W, Li S, Li J, Liu X, Bao M, Cui X, Yang J, Cui Q, Geng B, Cai J. AK098656, a Novel Vascular Smooth Muscle Cell-Dominant Long Noncoding RNA, Promotes Hypertension. Hypertension. 2018; 71:262–72. 10.1161/HYPERTENSIONAHA.117.09651 29279317

[25] Wang YN, Shan K, Yao MD, Yao J, Wang JJ, Li X, Liu B, Zhang YY, Ji Y, Jiang Q, Yan B. Long Noncoding RNA-GAS5: A Novel Regulator of Hypertension-Induced Vascular Remodeling. Hypertension. 2016; 68:736–48. 10.1161/HYPERTENSIONAHA.116.07259 27432865

[26] Shi L, Tian C, Sun L, Cao F, Meng Z. The lncRNA TUG1/miR-145-5p/FGF10 regulates proliferation and migration in VSMCs of hypertension. Biochem Biophys Res Commun. 2018; 501:688–95. 10.1016/j.bbrc.2018.05.049 29758198

[27] Lu C, Shi X, Wang AY, Tao Y, Wang Z, Huang C, Qiao Y, Hu H, Liu L. RNA-Seq profiling of circular RNAs in human laryngeal squamous cell carcinomas. Mol Cancer. 2018; 17:86. 10.1186/s12943-018-0833-x 29716593 PMC5930968

[28] Miao R, Wang Y, Wan J, Leng D, Gong J, Li J, Liang Y, Zhai Z, Yang Y. Microarray expression profile of circular RNAs in chronic thromboembolic pulmonary hypertension. Medicine (Baltimore). 2017; 96:e7354. 10.1097/MD.0000000000007354 28682884 PMC5502157

[29] Wu N, Jin L, Cai J. Profiling and bioinformatics analyses reveal differential circular RNA expression in hypertensive patients. Clin Exp Hypertens. 2017; 39:454–59. 10.1080/10641963.2016.1273944 28534714

[30] Bao X, Zheng S, Mao S, Gu T, Liu S, Sun J, Zhang L. A potential risk factor of essential hypertension in case-control study: circular RNA hsa_circ_0037911. Biochem Biophys Res Commun. 2018; 498:789–94. 10.1016/j.bbrc.2018.03.059 29526758

[31] Cohen ED, Tian Y, Morrisey EE. Wnt signaling: an essential regulator of cardiovascular differentiation, morphogenesis and progenitor self-renewal. Development. 2008; 135:789–98. 10.1242/dev.016865 18263841

[32] Foulquier S, Daskalopoulos EP, Lluri G, Hermans KC, Deb A, Blankesteijn WM. WNT signaling in cardiac and vascular disease. Pharmacol Rev. 2018; 70:68–141. 10.1124/pr.117.013896 29247129 PMC6040091

[33] Vallée A, Lévy BL, Blacher J. Interplay between the renin-angiotensin system, the canonical WNT/β-catenin pathway and PPARγ in hypertension. Curr Hypertens Rep. 2018; 20:62. 10.1007/s11906-018-0860-4 29884931

[34] Kim Y, Noren Hooten N, Dluzen DF, Martindale JL, Gorospe M, Evans MK. Posttranscriptional Regulation of the Inflammatory Marker C-Reactive Protein by the RNA-Binding Protein HuR and MicroRNA 637. Mol Cell Biol. 2015; 35:4212–21. 10.1128/MCB.00645-15 26438598 PMC4648813

[35] Li JX, Ding XM, Han S, Wang K, Jiao CY, Li XC. mir-637 inhibits the proliferation of cholangiocarcinoma cell QBC939 through interfering CTSB expression. Eur Rev Med Pharmacol Sci. 2018; 22:1265–76. 10.26355/eurrev_201803_14467 29565483

[36] Li YM, Liu XY. Molecular mechanisms underlying application of serum procalcitonin and stool miR-637 in prognosis of acute ischemic stroke. Am J Transl Res. 2016; 8:4242–49. 27830008 PMC5095317

[37] Jesus IC, Mesquita TR, Monteiro AL, Parreira AB, Santos AK, Coelho EL, Silva MM, Souza LA, Campagnole-Santos MJ, Santos RS, Guatimosim S. Alamandine enhances cardiomyocyte contractility in hypertensive rats through a nitric oxide-dependent activation of CaMKII. Am J Physiol Cell Physiol. 2020; 318:C740–50. [Epub ahead of print]. 10.1152/ajpcell.00153.2019 31913703 PMC7191420

[38] Jusic A, Devaux Y, and EU-CardioRNA COST Action (CA17129). Noncoding RNAs in Hypertension. Hypertension. 2019; 74:477–92. 10.1161/HYPERTENSIONAHA.119.13412 31352819 PMC6686966

[39] Joint Committee for Guideline Revision. 2018 Chinese Guidelines for Prevention and Treatment of Hypertension-A report of the Revision Committee of Chinese Guidelines for Prevention and Treatment of Hypertension. J Geriatr Cardiol. 2019; 16:182–241. 10.11909/j.issn.1671-5411.2019.03.014 31080465 PMC6500570

[40] Guo LL, Song CH, Wang P, Dai LP, Zhang JY, Wang KJ. Competing endogenous RNA networks and gastric cancer. World J Gastroenterol. 2015; 21:11680–87. 10.3748/wjg.v21.i41.11680 26556995 PMC4631969

[41] Prieto C, Risueño A, Fontanillo C, De las Rivas J. Human gene coexpression landscape: confident network derived from tissue transcriptomic profiles. PLoS One. 2008; 3:e3911. 10.1371/journal.pone.0003911 19081792 PMC2597745

