# Supplementary material for: Identification of candidate lncRNAs and circRNAs regulating WNT3/β-catenin signaling in essential hypertension
Source: Aging (Albany NY). 2020 May 11;12(9):8261–88. doi: 10.18632/aging.103137 (PMC7244030; doi:10.18632/aging.103137)
Supplement: Supplementary Figures [file aging-12-103137-s002..pdf]

SUPPLEMENTARY FIGURES

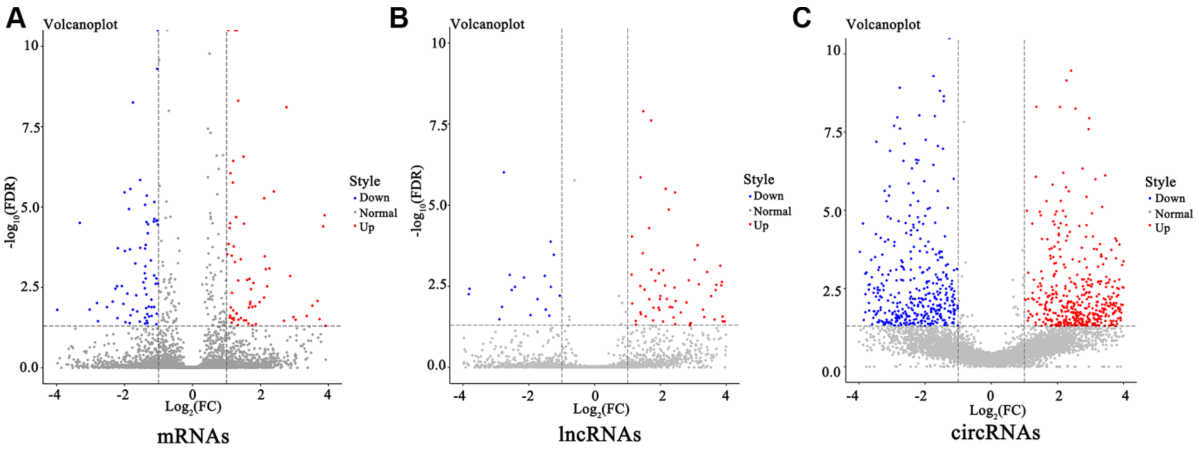

Supplementary Figure 1. All differentially expressed (DE) mRNAs (**A**) lncRNAs (**B**) and circRNAs (**C**) volcanic plots compared to the normotension. X-axis: The fold change is expressed as $\log_2$(FC); the Y-axis: *P* value is expressed as $-\log_{10}$(FDR). The reds dot in the figure indicates up. The blues dot indicates down.

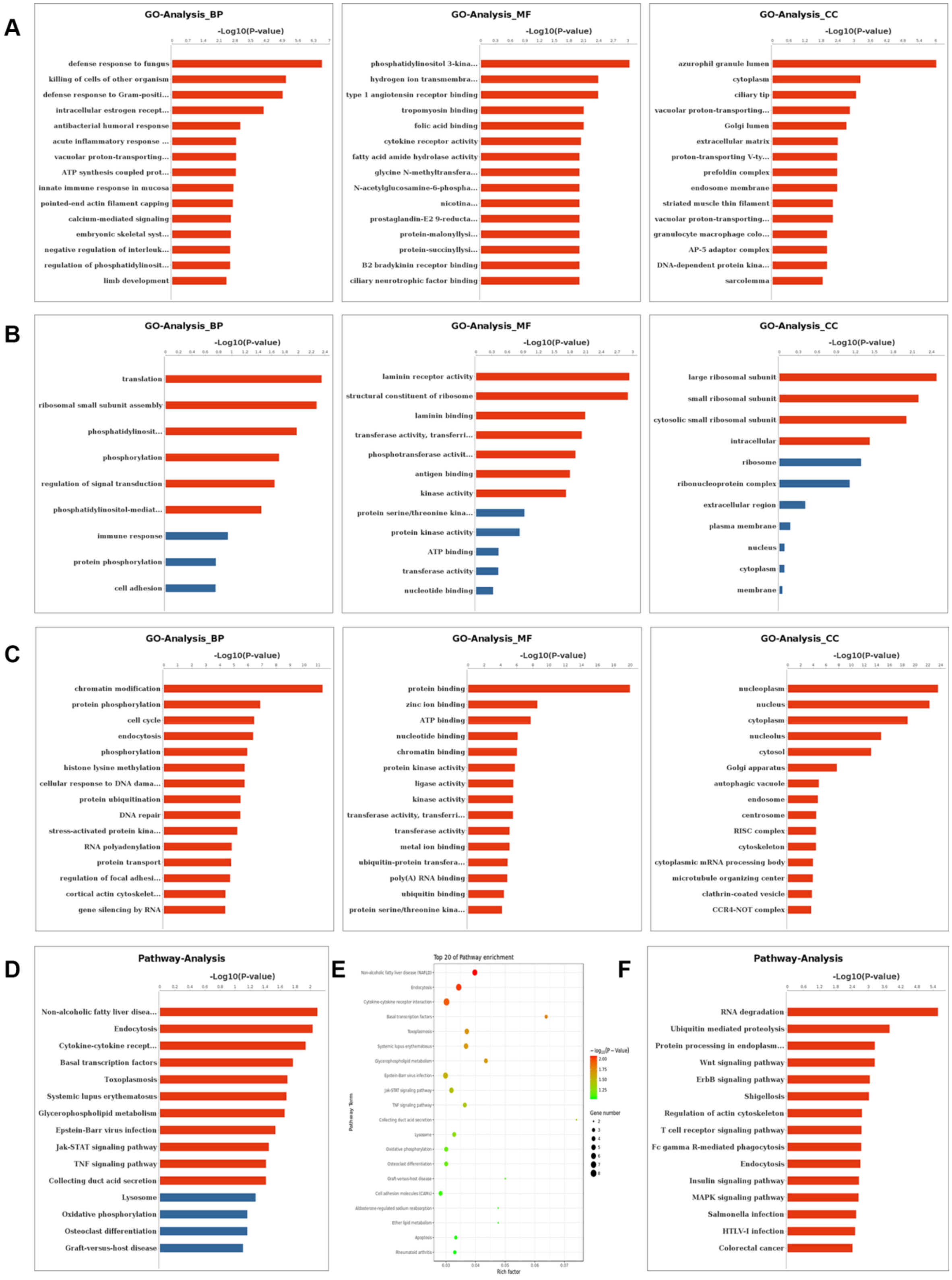

Supplementary Figure 2. Functional annotation of DE mRNAs, lncRNAs and circRNAs in hypertensive patients compared to the normotension. (**A**–**C**) Gene Ontology (GO) analysis of DE mRNAs, lnRNAs and circRNAs. Biological process (BP); Cellular Component (CC); Molecular Function (MF). (**D**) Kyoto Encyclopedia of Genes and Genomes (KEGG) pathway analysis of DE mRNAs and disease correlation. (**E**) KEGG pathway enrichment analyses for DE mRNAs. Node size: gene ratio, node color: *P* value. (**F**) KEGG pathway analysis of DE circRNAs. The red column represents *P*<0.05 and the blue column represents *P*>0.05.

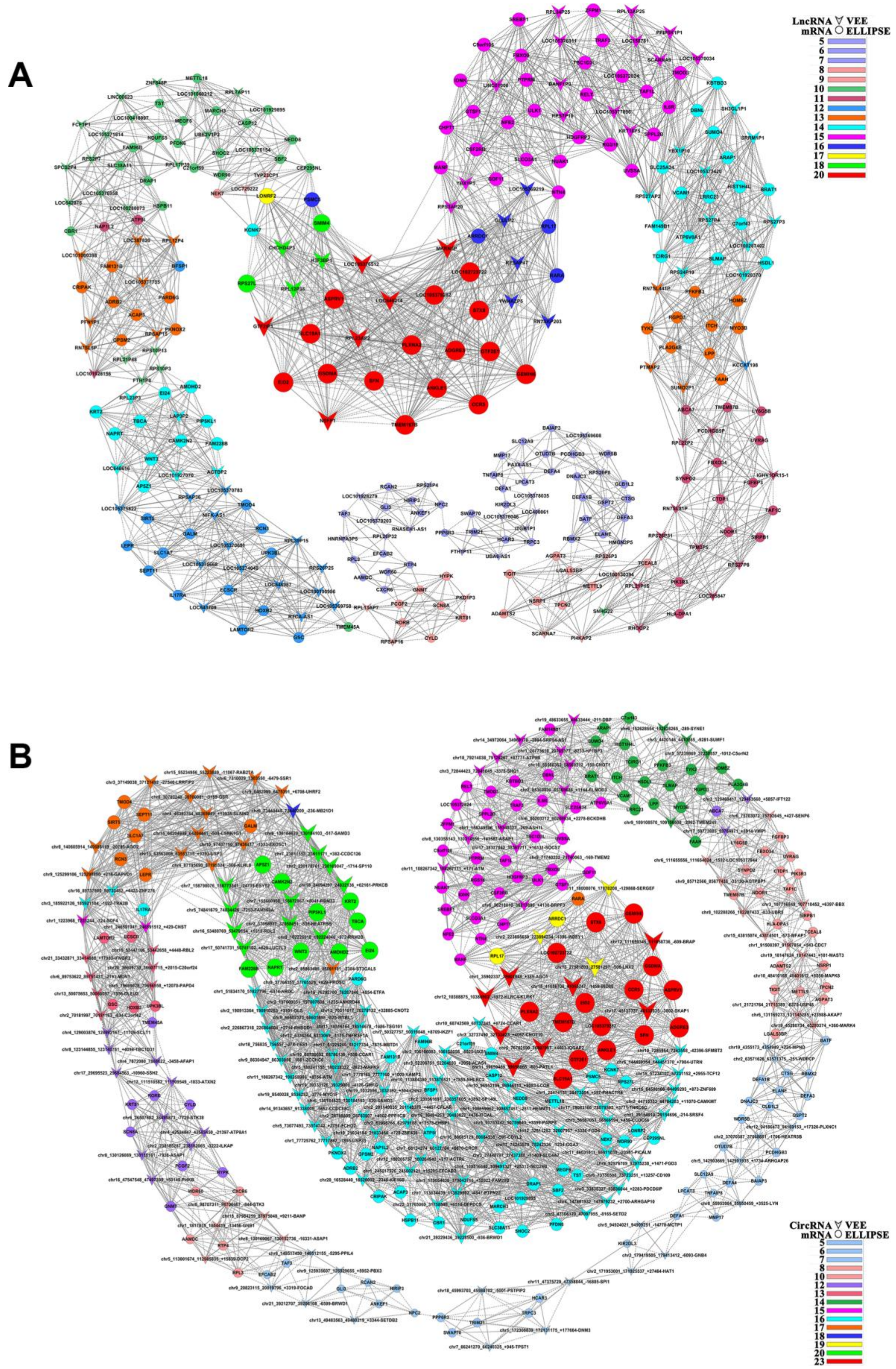

Supplementary Figure 3. Co-expression network. (**A**) Co-expression network of DE lncRNAs and mRNAs. (**B**) DE circRNAs and mRNAs co-expression network (GRCh38). Red nodes represent the key regulatory genes with the highest k-core values. Node colors indicate k-core values. Node size represents the k-core power, and the distance between two nodes represents the interaction between the two RNAs. The solid line in the figure is a positive correlation and the dotted line represents a negative correlation.

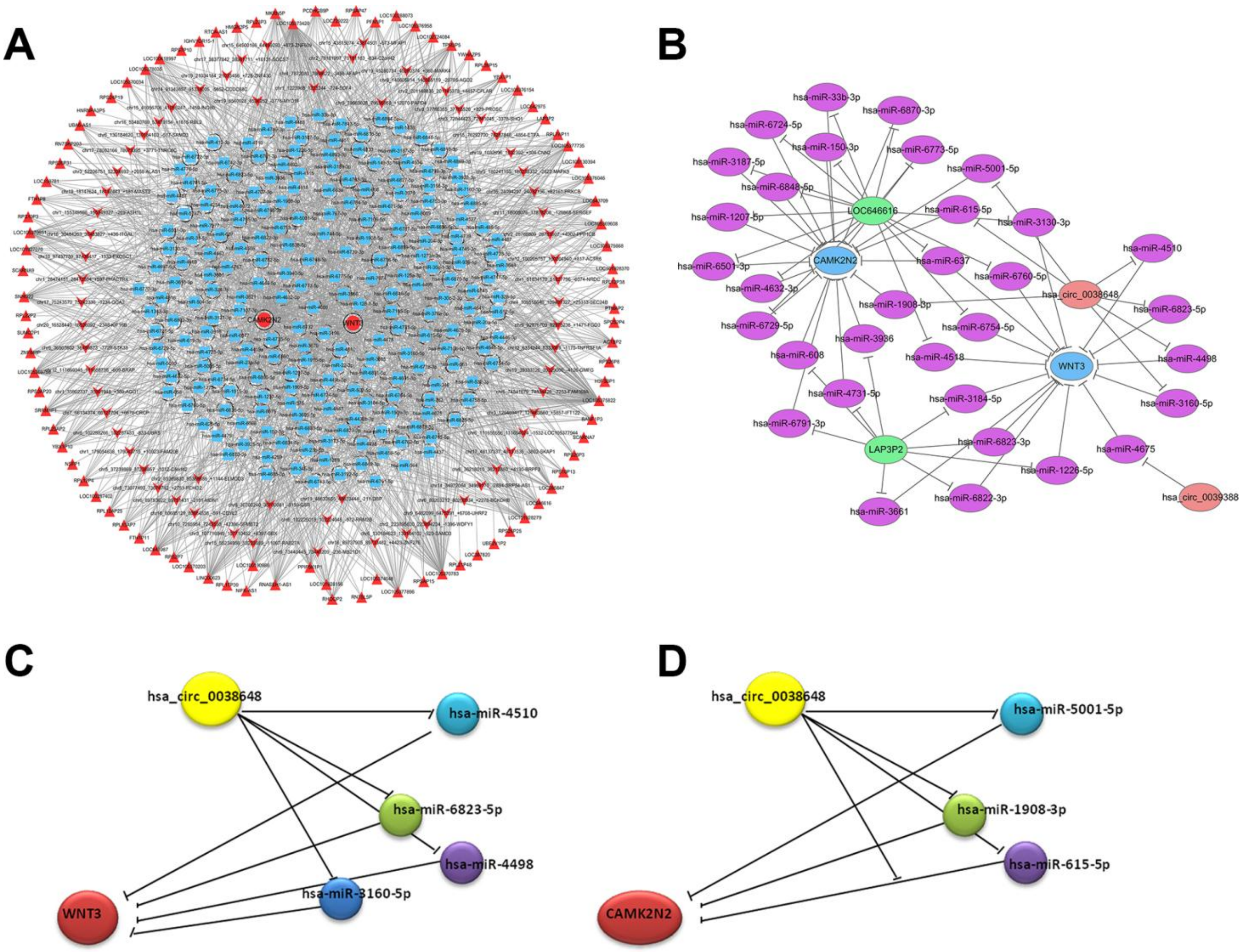

Supplementary Figure 4. (**A**) WNT3 and CAMK2N2 related micRNA-based sponge regulatory network. Triangles represent DE lncRNAs, vee represents DE circRNAs and squares represent miRNAs. (**B**) sub-networks of the most significant DE lncRNAs and circRNAs with WNT3 and CAMK2N2. (**C**, **D**) A miRNA-based sponge regulatory subnetwork depicting hsa_circ_0038648/miRNA/WNT3 and hsa_circ_0038648/miRNA/CAMK2N2 interactive modules.

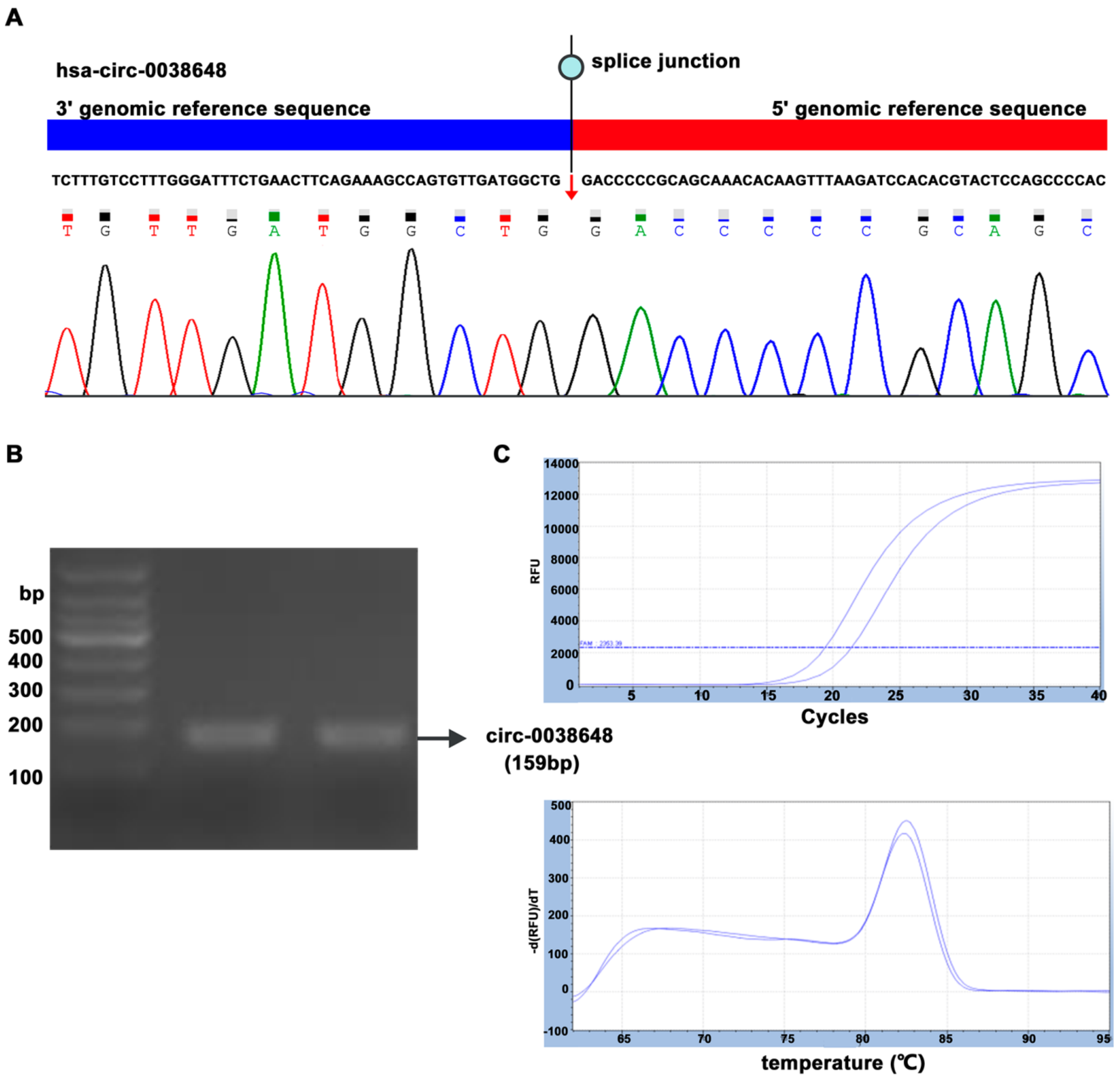

Supplementary Figure 5. (**A**) The sequence of hsa_circ_0038648 (upper panel) was consistent with the result of Sanger sequencing (lower panel). The red arrow indicates the backsplice site. (**B**) The specific primers of hsa_circ_0038648 were validated by agarose gel electrophoresis. (**C**) Amplification curve and melting curve of hsa_circ_0038648 sample.

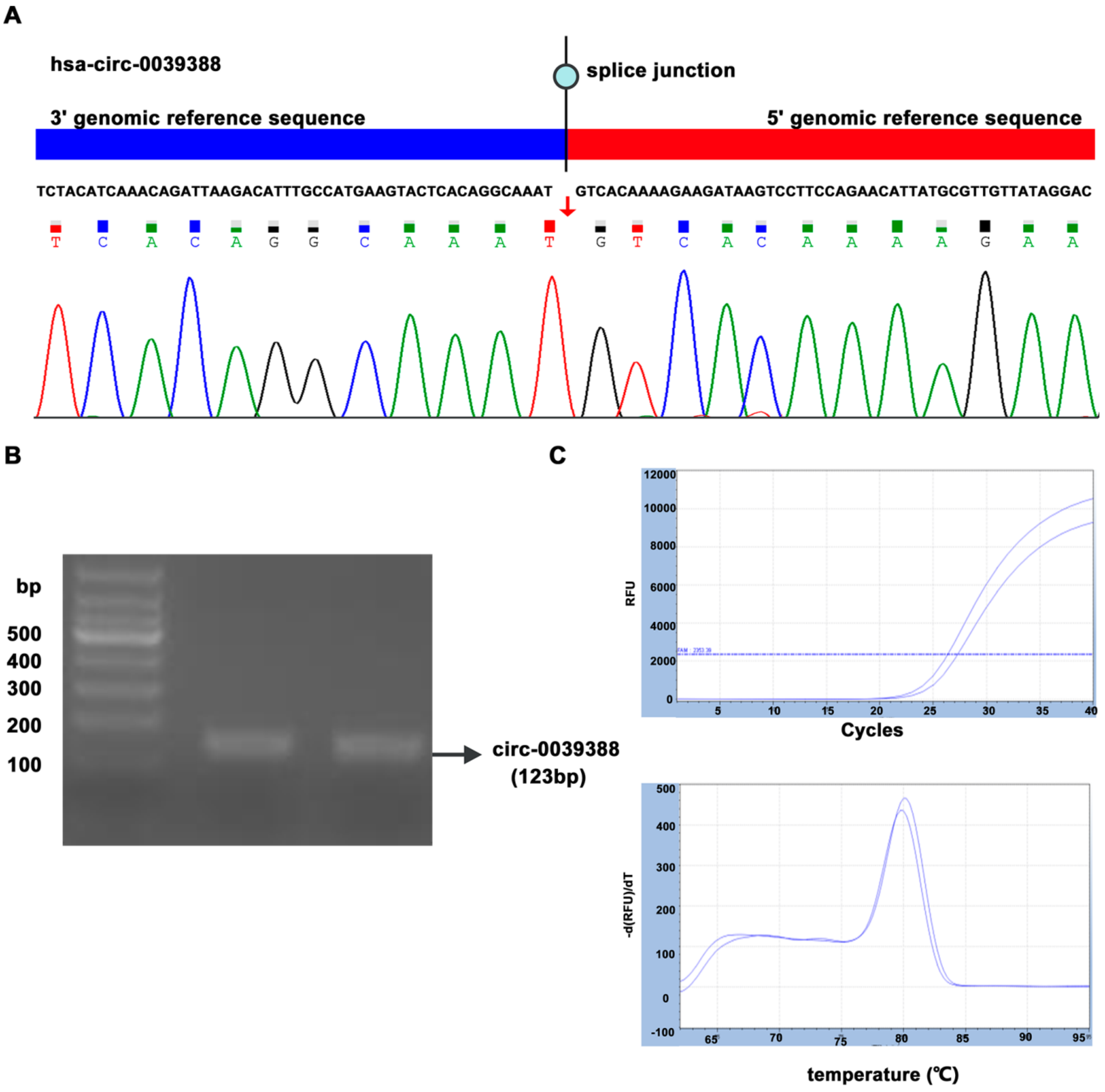

Supplementary Figure 6. (**A**) The sequence of hsa_circ_0039388 (upper panel) was consistent with the result of Sanger sequencing (lower panel). The red arrow indicates the backsplice site. (**B**) The specific primers of hsa_circ_0039388 were validated by agarose gel electrophoresis. (**C**) Amplification curve and melting curve of hsa_circ_0039388 sample.

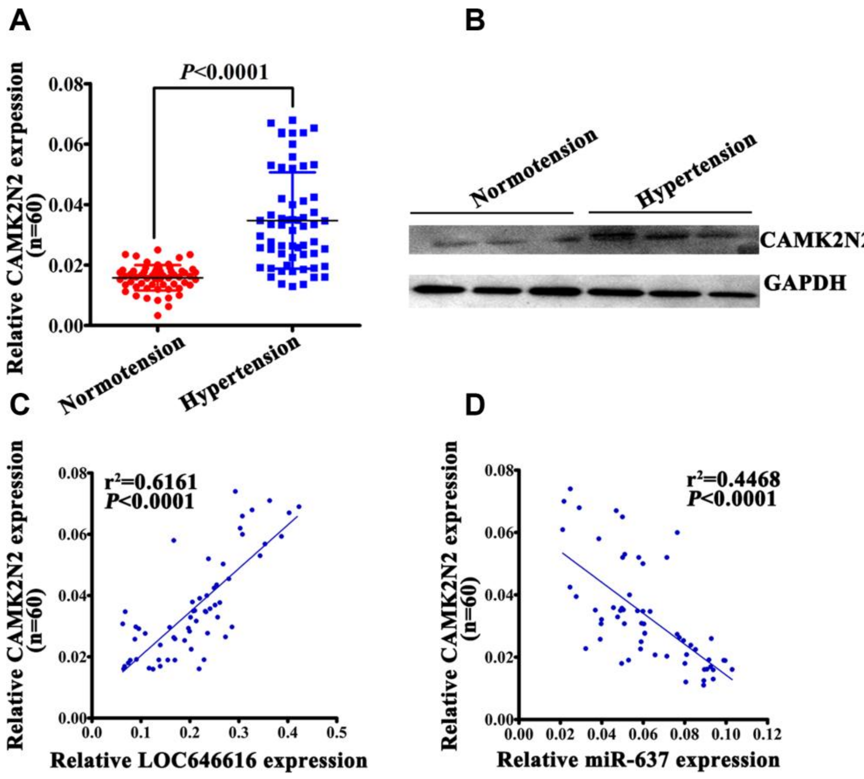

Supplementary Figure 7. (**A**, **B**) Relative mRNA and protein levels of CAMK2N2 in PBMNCs of hypertensive subjects and normotensive controls. Bivariate correlation analysis demonstrated that CAMK2N2 was positively correlated with LOC646616 (**C**) and inversely correlated with miR-637 (**D**) in the same set of patients.

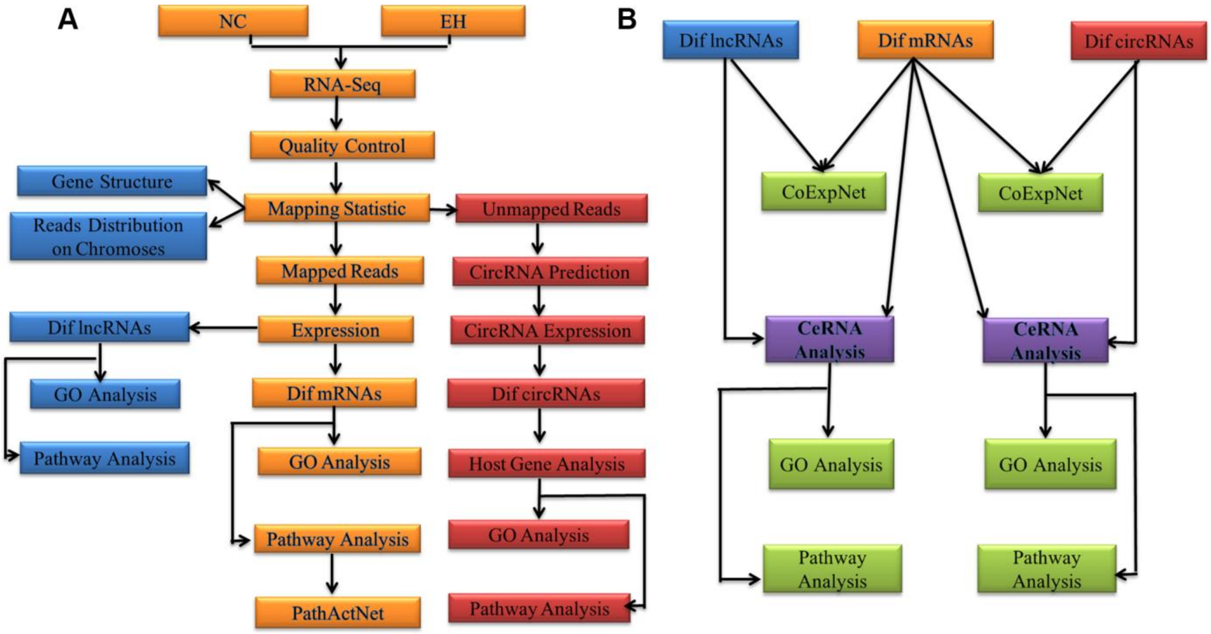

Supplementary Figure 8. Comprehensive analyses workflow. (**A**) Comprehensive analyses workflow of lncRNAs, mRNAs, and circRNAs in hypertension and normal controls. (**B**) Workflow for comprehensive joint analysis between DE lncRNAs, mRNAs and circRNAs.
