# Supplementary material for: Identification of candidate lncRNAs and circRNAs regulating WNT3/β-catenin signaling in essential hypertension
Source: Aging (Albany NY). 2020 May 11;12(9):8261–88. doi: 10.18632/aging.103137 (PMC7244030; doi:10.18632/aging.103137)
Supplement: Supplementary Tables [file aging-12-103137-s001..pdf]

SUPPLEMENTARY TABLES

Supplementary Table 1. Characteristics of 3 hypertensive patients and 5 normotensive individuals of sequencing.

Characteristics<br>Gender (M/F)	Hypertension<br>(n=3) 1/2	Normotension<br>(n=5) 3/2
Age (years)	59.73 ± 6.08	60.17 ± 4.10
SBP (mmHg)	161.00 ± 15.10**	114.70 ± 7.40
DBP (mmHg)	98.77 ± 5.40**	78.97 ± 6.16
BMI (kg/m^2)	25.81 ± 3.29	24.17 ± 2.30
FBG (mmol/L)	5.36 ± 1.04	5.08 ± 1.16
TG (mmol/L)	1.67± 1.09	1.17 ± 0.86
LDL (mmol/L)	2.97 ± 0.91	2.80 ± 0.73
HDL (mmol/L)	1.14 ± 0.12	1.32 ± 0.27
CHO (mmol/L)	4.93 ± 1.02	4.71 ± 0.79

#Data are mean ±standard deviation (SD).
M, male; F, female; SBP, systolic blood pressure; DBP, diastolic blood pressure; BMI, body mass index; FBG, fasting blood glucose; TG, triglycerides; LDL, low density lipoprotein; HDL, high density lipoprotein; CHO, cholesterol.
***P*<0.01 vs. normotensive individuals.

Supplementary Table 2. Correlation between the most significant DE lncRNAs, circRNAs and mRNAs.

Name	mRNA	Pearson	P-value	FDR
LOC646616	WNT3	0.999888	0.009524	0.048855
	CAMK2N2	0.999920	0.008051	0.047351
LAP3P2	WNT3	0.999691	0.015827	0.048855
	CAMK2N2	0.999996	0.001748	0.044093
hsa_circ_0039388	WNT3	0.999898	0.009100	0.045505
	CAMK2N2	0.999122	0.026675	0.048718
hsa_circ_0038648	WNT3	0.999998	0.001173	0.041120
	CAMK2N2	0.999668	0.016402	0.048514
WNT3	CAMK2N2	0.999619	0.017575	0.048855

#FDR: false discovery rate.

Supplementary Table 3. Expression data (in units of FPKM) for the most significant DE lncRNAs and circRNAs.

	FPKM	FPKM	FPKM	FPKM	FPKM	FPKM	FPKM	FPKM	EH1/2/3 vs NC1/2/3/4/5	
gene type	EH_1F	EH_1M	EH_2F	NC_1M	NC_2F	NC_2M	NC_3M	NC_3F	EH average	NC average
LOC646616	23.32145695	22.82128303	19.68736046	1.43010615	1.863003619	0.90103621	1.722328318	1.744558523	21.94336681	1.532206564
LAP3P2	0.916949814	0.268886904	0.451702508	0	0.177244944	0	0	0	0.5458464	0.035449
circ_0039388	7.792171867	1.559141998	3.09035545	0	0	0	0.270651619	0	4.147223105	0.054130324
circ_0038648	3.896085934	1.133921453	1.85421327	0.116586733	0.117713797	0	0	0	2.29474	0.04686

Supplementary Table 4. Characteristics of 60 hypertensive patients and 60 normotensive individuals.

Characteristics<br>Gender (M/F)	Hypertension<br>(n=60) 26/34	Normotension<br>(n=60) 30/30
Age (years)	54.33± 8.94	53.37± 9.18
SBP (mmHg)	158.33± 13.16**	119.60± 9.59
DBP (mmHg)	96.20± 5.46**	79.53± 5.75
Family history (%)	36.67	26.67
Smoking (%)	43.33	46.67
Drinking (%)	53.33	50.00
BMI (kg/m^2)	26.29±3.21	25.39±2.81
FBG (mmol/L)	5.16±0.64	4.99±0.51
TC (mmol/L)	4.57±0.98*	4.07±0.60
TG (mmol/L)	1.87±0.14	1.55±0.09
HDL (mmol/L)	1.06±0.22	1.16±0.19
LDL (mmol/L)	2.81±0.90	2.58±0.78

#Data are mean ± SD.
**P*<0.05, ***P* <0.01 vs. normotensive individuals.

Supplementary Table 5. Whole gene sequencing results of 7 DE lncRNAs and 2 DE cicRNAs.

Test_id	EH	NH	Log_2 FC	FDR	Style	Length
DE lncRNAs						
SCARNA7	12882.37	338.65	5.25	0.000072	up	329
RPS3AP20	223.78	9.59	4.55	0.009161	up	858
LOC646616	13.56	0.92	3.89	0.038099	up	1346
RPL34P25	7.39	39.60	-2.42	0.003287	down	436
RPSAP16	31.19	64.98	-1.06	0.006135	down	986
RPSAP36	6.47	24.99	-1.95	0.024000	down	1010
LAP3P2	12.83	0.75	4.09	0.006210	up	1695
DE circRNAs						
hsa_circ_0039388	26.09	1.06	4.63	0.000000	up	381
hsa_circ_0038648	15.90	0.79	4.33	0.000000	up	533

*The screening criteria were log_2 FC >1 or <-1, FDR<0.05.

Supplementary Table 6. Multiple stepwise regression analysis showing variables independently associated with LOC646616 and hsa_circ_0038648.

	β	SE	β'	t
LOC646616				
Constant	-0.280	0.276		-0.101
SBP	0.345	0.098	0.421	3.525*
Cys C	0.262	0.123	0.230	2.135*
Hcy	0.317	0.154	0.250	2.051*
hsa_circ_0038648				
Constant	0.162	0.416		0.390
SBP	0.300	0.142	0.364	2.119*
HDL	-0.330	0.161	-0.233	-2.039*

#β': standardized β. Variables included in the original model are SBP, DBP, BMI, FBG, TC, TG, HDL, LDL, CRP, Hcy and Cys C. CRP, C-reactive protein; Hcy, homocysteine; Cys C, cystatin C. **P*<0.05.

Supplementary Table 7. Primers sequences used for the studies.

Test_id	Primer
RPSAP36	F primer 5'-GCTGAAGACCAGCCTCTCATA-3'
	R primer 5'-CCACATCAAACCCACGGAGT-3'
LOC646616	F primer 5'-ACCGAAGGCACTACACGC-3'
	R primer 5'-GAGATTGGAGCAGCAAATGG-3'
RPS3AP20	F primer 5'-TTTGAATTGGGAAAGCTCATG-3'
	R primer 5'-AACAGATTCTTGGACTGGTGG-3'
RPL34P25	F primer 5'-TCCCAGACCCTTGGTAATAGA-3'
	R primer 5'-ACATGCCACATGCAGATTTT-3'
SCARNA7	F primer 5'-GCTTGTGGTGGCTATGGAA-3'
	R primer 5'-GGTGAGCTGTTCTTGGGTTAT-3'
RPSAP16	F primer 5'-GGGTGCCCTCTGTGCCTAT-3'
	R primer 5'-CCTGAGCAGTGGGAGCTGTA-3'
LAP3P2	F primer 5'-GAGCCCTCAGTCTTCTTG -3'
	R primer 5'-AGATACCACCACTGTCAAA -3'
GAPDH	F primer 5'-TCTCTGCTCCTCCTGTTCGA-3'
	R primer 5'- GCGCCCAATACGACCAAATC-3'
hsa_circ_0039388	F primer 5'-AGGGGAGACCTCATTCAGTTCTACA -3'
	R primer 5'-TGTAGAACTGAATGAGGTCTCCCCT-3'
hsa_circ_0038648	F primer 5'-TTTTTCTCTATGCAGTCAGCTGAAA -3'
	R primer 5'-TTTCAGCTGACTGCATAGAGAAAAA-3'
miR-637	F primer 5'-ACACTCCAGCTGGGACTGGGGGCTTTCGGGCT -3'
	R primer 5'-CTCAACTGGTGTCGTGGAGTCGGCAATTCAGTTGAGACGCAGAG-3'
U6	F primer 5'-CTCGCTTCGGCAGCACA-3'
	R primer 5'-AACGCTTCACGAATTTGCGT-3'
miR-637 mimics	Sense 5'-ACUGGGGGCUUUCGGGCUCUGCGU-3'
	Antisense 5'-GCAGAGCCCGAAAGCCCCCAGUUU-3'
miR-637 inhibitor	Sense 5'ACGCAGAGCCCGAAAGCCCCCAGU-3'
WNT3	F primer 5'- CTGCCAGGAGTGTATTCGCATC -3'
	R primer 5'- GAGAGCCTCCCCGTCCACAG -3'
CAMK2N2	F primer 5'- CGCAGACCCCGAAGGTT-3'
	R primer 5'-TTGCCAGCGAAGAAGGAGTT-3'
β-catenin	F primer 5'-GGGACACAGCAGCAATTTGT-3'
	R primer 5'-CAGCTGCACAAACAATGGAA-3'

* RPSAP36, ribosomal protein SA pseudogene 36; LOC646616 transmembrane protein 183A pseudogene; RPS3AP20, ribosomal protein S3a pseudogene 20; RPL34P25, ribosomal protein L34 pseudogene 25; SCARNA7, small Cajal body-specific RNA 7; RPSAP16, ribosomal protein SA pseudogene 16; LAP3P2, leucine aminopeptidase 3 pseudogene 2; GAPDH, glyceraldehyde-3-phosphate dehydrogenase; has_circ_0039388, Homo sapiens_circular RNA_0039388; hsa_circ_0038648, Homo sapiens_circular RNA_0038648; miR-637, microRNA-637; U6, U6 small nuclear RNA; WNT3, Wnt family member 3; CAMK2N2, calcium/calmodulin dependent protein kinase II inhibitor 2.

Supplementary Table 8. siRNA sequences used for the studies.

	Sequence(5'-3')
LOC646616-1	Sense GCGGCUGGAUUAUAUAUCUTT
	Antisense AGAUAUAUAAUCCAGCCGCTT
LOC646616-2	Sense CCAUAUAUGUAUGAGCCAUTT
	Antisense AUGGCUCAUACAUAUAUGGTT
LOC646616-3	Sense CAGGAACCAAUGUGGAAAUTT
	Antisense AUUUCCACAUUGGUUCCUGTT
